# Retrospective Validation of a Resource-Aware Assistive AI Tool for Screening Prostate Needle Biopsies and Classifying Prostate Adenocarcinoma into ISUP Grade Groups

**DOI:** 10.3390/life16081257

**Published:** 2026-07-29

**Authors:** Sahil Ajit Saraf, Wai Po Kevin Teng, Kolangara Veetil Santosh, Sencer Karakaya, Amala Abbas, Tony Kiat Hon Lim, Kankanamage Malinda Amesh Karasinghe, Zachariah Chowdhury, Priti Singh, Anubhav Narwal, Samrat Bhattacharjee, Bidyut Bikash Gogoi, Bahoran Singh, Mohamid Afroz Khan, Daljeet Kaur, Kaveh Taghipour, Li Yan Khor

**Affiliations:** 1 Department of Anatomical Pathology, Singapore General Hospital, 20 College Road, Singapore 169856, Singapore; lim.kiat.hon@singhealth.com.sg (T.K.H.L.); karasinghe.malinda@singhealth.com.sg (K.M.A.K.); khor.li.yan@singhealth.com.sg (L.Y.K.); 2 Department of Medical Affairs, Qritive Pte. Ltd., 71 Ayer Rajah Crescent, #04-09, Singapore 139951, Singapore; amala.abbas@qritive.com; 3 Artificial Intelligence Unit, Qritive Pte. Ltd., Singapore 139951, Singapore; kevin.teng@qritive.com (W.P.K.T.); karakayasencers@gmail.com (S.K.); kaveh@qritive.com (K.T.); 4 Department of Histopathology, Metropolis Healthcare Ltd., Bengaluru 560003, India; santoshpath@yahoo.com; 5 Duke NUS Medical School, Singapore 169857, Singapore; 6 Department of Oncopathology, Mahamana Pandit Madan Mohan Malviya Cancer Centre, Homi Bhabha National Institute, Varanasi 221005, India; oncopath.tmhv@gmail.com; 7 Department of Pathology, Lady Hardinge Medical College, New Delhi 110001, India; doctorpriti@gmail.com; 8 Department of Pathology, All India Institute of Medical Sciences, New Delhi 110029, India; anubhavnarwal88@gmail.com; 9 Department of Pathology, Lakhimpur Medical College & Hospital, North Lakhimpur 787051, India; itsdrsam@gmail.com; 10 Department of Oncopathology, Gujarat Cancer and Research Institute, Ahmedabad 380016, India; drgogoibidyut@gmail.com; 11 Department of Lab Services, BIMR Hospital, Gwalior 474005, India; bahoran.raj@gmail.com; 12 Department of Laboratory Medicine and Blood Bank, Kokilaben Dhirubhai Ambani Hospital, Indore 452010, India; faiz30aug@yahoo.com; 13 Department of Pathology, Gauhati Medical College & Hospital, Guwahati 781032, India; doctordaljeetpath@gmail.com

**Keywords:** artificial intelligence, prostate adenocarcinoma, prostate needle biopsy, AI workflows, digital pathology

## Abstract

**Introduction:** Prostate needle biopsy reporting is time-consuming because each case typically includes 12–18 cores that require detailed assessment before a final case-level diagnosis is issued. Reporting is also influenced by pathologist expertise, particularly for tumour detection and ISUP Grade Group assignment. This study evaluated an artificial intelligence (AI)-based system designed to identify tumour and provide segmentation-based visual outputs highlighting Gleason patterns and suggesting an ISUP Grade Group. **Methods:** A strongly supervised approach was used to develop the algorithm. Twenty-seven pathologists annotated 2115 prostate needle biopsy whole-slide images, followed by two levels of senior pathologist reviews. An independent external test dataset of 150 prostate needle biopsy whole-slide images was then evaluated. Ground truth (GT) was established by two genitourinary pathologists, with disagreements resolved by consensus. The same slides were independently reviewed by 11 pathologists without AI assistance, while the AI system analysed the slides in parallel. AI performance and pathologist consensus were compared to GT. **Results:** For benign versus malignant classification, the AI identified all 109 malignant slides, with no false-negative predictions, while the pathologist consensus missed two malignant slides. The AI correctly classified 38/41 benign slides, compared to 39/41 for the pathologist consensus. For ISUP Grade Group assignment, the AI showed exact agreement with GT in 57/109 malignant cases. The AI more frequently assigned a higher Grade Group than GT. **Conclusions:** In this retrospective evaluation, the AI system showed high sensitivity for tumour detection and promising ordinal agreement for ISUP Grade Group assignment. These findings support further evaluation of the system within supervised prostate biopsy workflows.

## 1. Introduction

The Gleason score, currently reported as the International Society of Urological Pathology (ISUP) Grade Group (GG), is a central determinant of clinical decision-making in prostate cancer and represents one of the most important histopathological parameters derived from prostate biopsy specimens [1]. The probability of cancer detection increases with the volume of tissue examined [2]; consequently, contemporary prostate needle biopsy protocols typically include 12–18 cores per case to ensure adequate sampling of the different anatomical zones of the prostate. Comprehensive evaluation of these specimens is labour-intensive, requiring the detailed assessment of each core before the synthesis of a case-level diagnosis, with reporting times ranging from 20 min to more than one hour per case [3].

Despite its clinical importance, prostate biopsy reporting remains inherently subjective and is highly dependent on pathologist expertise, resulting in substantial inter- and intra-observer variability [4]. In a study involving the central pathology analysis of 96 prostate biopsies, complete concordance was observed in only 72% of cases. Among discordant cases, 75% were downgraded and 25% were upgraded, with most discrepancies involving the distinction between Gleason patterns 3 and 4 [5]. Variability is further compounded when estimating tumour extent. In a separate study assessing tumour percentage estimation using virtual microscopy, 304 pathologists reported tumour extent using different metrics, including percentages, millimetres, or a combination of both [6]. These methodological differences contributed to marked variability in reported tumour burden.

Artificial intelligence (AI) has shown potential to improve diagnostic precision, reproducibility, and workflow efficiency in pathology. For example, a 2024 study reported that AI can enhance diagnostic precision and efficiency by automating Ki-67 scoring [7]. Similarly, AI may support diagnostic workflows in prostate pathology, particularly when used as a decision-support tool alongside expert pathologist interpretation [8]. By enabling more systematic evaluation of larger tissue volumes, optimised AI systems have the potential to contribute to improved tumour detection sensitivity and create a greater consistency in Gleason grading [9].

As a step toward exploring AI-supported prostate biopsy assessment, we developed a deep learning-based model that identifies tumour regions and provides a suggested ISUP GG for review. The model is intended as an assistive tool for pathologists and generates segmentation-based visual outputs highlighting individual Gleason patterns, thereby supporting transparent interpretation and facilitating an informed final diagnostic decision.

Importantly, this AI model was developed in the context of a developing country, where access to infrastructure, specialist expertise, large-scale annotated datasets, automated laboratory systems, and high-cost computational resources may be more limited than in developed-country settings. The development and evaluation of such a model under these conditions provides practical insight into the challenges and opportunities associated with building AI tools in resource-constrained environments. This experience may be valuable for other AI developers and pathology groups working under similar circumstances, particularly in regions where digital pathology and AI adoption are still emerging but where the need for scalable diagnostic support is substantial.

## 2. Methods

### 2.1. Training Dataset

Annotation quality is critical to the performance of supervised learning algorithms. To develop a model that approximates routine diagnostic workflows, a strongly supervised annotation strategy was employed. A total of 2115 prostate needle biopsy whole-slide images (WSIs) were used for algorithm development and annotated by 27 pathologists. These slides were partitioned into training, validation, and internal test subsets, as described below. In addition, a separate external evaluation cohort of 150 prostate needle biopsy WSIs was selected for final performance assessment. All slides used in the study, including the algorithm-development dataset and the external evaluation cohort, were sourced through 64 Codon and comprised material from multiple participating institutions in India.

The slides were prepared from formalin-fixed, paraffin-embedded (FFPE) tissue blocks, stained with haematoxylin and eosin (H&E), and scanned at 40× magnification using a Grundium Ocus 40 scanner manufactured by Grundium, Saavutustenkatu 3, 33720 Tampere, Finland. The use of a single scanner provided consistency in image acquisition, but it also limits conclusions regarding scanner-level generalisability. Therefore, the findings should be interpreted as evidence of model performance under the scanning conditions used in this study, rather than as definitive proof of performance across all digital pathology platforms. The inclusion and exclusion criteria were applied uniformly across the algorithm-development and external evaluation datasets, as summarised in Table 1 and Table 2.

The dataset was selected to reflect the types of slides that may be encountered in routine histopathology practice, while maintaining sufficient diagnostic quality for model development and evaluation. The participating institutions contributed sequential prostate core needle biopsy cases collected between January 2021 and January 2022. All cases were anonymised in accordance with the applicable Institutional Review Board approval and were sourced using consistent study protocols. To reduce case-level redundancy, no more than two slides were included from any single needle biopsy case.

Only FFPE prostate core needle biopsy tissue sections stained with H&E were included in the study. Special stains, cytology preparations, non-prostate needle biopsy specimens, and irrelevant or non-representative tissue samples were excluded. Whole-slide images (WSIs) were scanned at 40× magnification and were required to have adequate resolution and sufficient image quality to permit diagnostic interpretation.

The slide selection criteria were intentionally pragmatic. Any slide deemed diagnostically interpretable by a pathologist was eligible for inclusion, provided that sufficient tissue was present to render a diagnosis. Slides showing minor artefacts, including tissue folding, staining variation, small scanning imperfections, or other limited technical issues, were included when these changes did not preclude diagnostic assessment (Table 1). This approach was adopted to avoid creating an artificially idealised dataset and to better reflect the variability encountered in routine practice where automation is not widespread.

All slides were manually stained because automated staining systems were not available at the participating institutions. Consequently, variability in H&E staining intensity, contrast, and colour balance was present across the dataset. This variability was permitted when tissue morphology remained interpretable. While this introduced pre-analytical heterogeneity, it also reflected the practical conditions under which histopathology services may operate in resource-limited settings. The study provides useful experience for AI development in developing-country environments, where access to automated laboratory infrastructure, subspecialist expertise, large-scale annotated datasets, and high-cost computational resources may be more limited than in highly resourced settings.

Slides were excluded if they were considered non-diagnostic or unsuitable for reliable interpretation. Exclusion criteria included insufficient tissue, poor preservation, extensive tissue folding, tearing, crushing, air bubbles obscuring key regions, severe over- or under-staining that impaired morphology, or other artefacts that precluded diagnosis (Table 2). WSIs with major scanning or technical issues, including extensive out-of-focus regions, incomplete scans, missing regions of interest, unreadable file formats, or file corruption, were also excluded. Duplicate slides from the same block were excluded when they did not provide additional analytical value.

Overall, the inclusion and exclusion criteria were designed to balance diagnostic quality with real-world representativeness. Slides were not excluded solely because of minor artefacts, staining variability, or limited scanning imperfections, provided that a pathologist could still render a diagnosis. This strategy supported the development and evaluation of the AI model on material that approximated routine histopathology practice in the participating centres. Quality checks were performed by pathologists and technical staff at both 64 Codon located at Door No. 41/708, JB Plaza, Ground Floor, Padivattom, Cochin, Kerala, 682024, India.and Qritive Pte. Ltd. located at 71 Ayer Rajah Crescent, #04-09, Singapore 139951.; at Qritive, the assessment was conducted by a team comprising three pathologists and one technical staff member.

### 2.2. Annotation Strategy

The first round of annotations was performed by pathologists with 3–5 years of diagnostic experience and subsequently underwent two levels of oversight. The first review was conducted by pathologists with more than 10 years of diagnostic experience, followed by final review and approval by two genitourinary (GU) pathology specialists. Only slides that passed this scrutiny were authorised for use in algorithm development.

All annotations were performed using the Qritive Pantheon v1.0 software platform. In total, 469,577 annotations were generated across the algorithm-development dataset, which comprised training, validation, and internal test subsets. These annotations included multiple histological classes: benign tissue, Gleason pattern 3, Gleason pattern 4, Gleason pattern 5, damaged tissue, blood vessels, inflammation, stroma, nerves, and perineural invasion (PNI). Details of the dataset split (Table 3) and model development are described in Section 2.4, “Algorithm Training and Model Architecture.”

Certain labels such as inflammation, blood vessels, stroma, nerves, damaged tissue, and PNI, were incorporated to provide histological context and to help reduce false-positive tumour predictions during model development. Inflammation was defined as any aggregation of inflammatory cells. Because lymphoid aggregates represented one of the recognised sources of false-positive detections, particularly when misclassified as high-grade tumour, particular attention was given to annotating lymphocytic aggregates, including small clusters. Other inflammatory cell collections, including neutrophilic aggregates, macrophages, and eosinophils, were also included under the inflammation label when present.

Necrotic tissue was annotated within the stroma category because the primary objective at this stage of development was to reduce false-positive tumour classification. Although stromal annotations may have contributed to improved contextual discrimination, the limited number of necrotic examples precluded reliable training for necrosis-specific detection.

Intraductal carcinoma was annotated as Gleason pattern 4 for the development of the current algorithm. Due to this, the current version does not provide a separate output for intraductal carcinoma. This distinction remains important because specific recognition and reporting of intraductal carcinoma may have clinical relevance and must continue to be assessed by the reporting pathologist.

High-grade prostatic intraepithelial neoplasia (HGPIN) and atypical small acinar proliferation (ASAP) were annotated as Gleason pattern 3 to prioritise sensitivity during algorithm development. This approach reflected the intended use of the model: to flag suspicious foci for review, not to issue a diagnosis independently. We acknowledge that this annotation strategy may increase the likelihood of false-positive tumour predictions in AI outputs. During model development, avoiding false-negative tumour detection was considered an important performance priority, particularly in a screening-oriented workflow.

Because routine IHC was not incorporated into annotation or ground-truth development, small atypical glands, borderline foci, HGPIN, ASAP, and benign mimics remain potential sources of uncertainty. This limitation is particularly relevant in settings where ancillary stains may be unavailable, inconsistently performed, or reserved for selected equivocal cases.

AI outputs were treated as review prompts rather than final diagnostic labels. Accordingly, a limited increase in false-positive tumour detections was considered acceptable during development, provided that such outputs could be reviewed and adjudicated by a pathologist. A false-positive rate of no more than 5% of the total cases was predefined as a practical development benchmark. In this experiment, the observed false-positive rate was 7.3%, marginally exceeding this threshold. Detailed performance metrics are presented in Section 3.3, “Benign versus Malignant Classification Performance.”

### 2.3. Test Dataset Selection and Study Protocol

An independent external test dataset comprising 150 prostate needle biopsy whole-slide images (WSIs) was selected for final evaluation of the AI system. As described in Section 2.1, all slides were sourced through the 64 Codon biobank and represented material contributed by multiple participating institutions. Similarly, to the algorithm-development dataset, all specimens were prepared from formalin-fixed, paraffin-embedded (FFPE) tissue blocks, stained manually with haematoxylin and eosin (H&E), and digitised at 40× magnification using a Grundium Ocus 40 whole-slide scanner (Grundium, Tampere, Finland). The inclusion and exclusion criteria applied to the test dataset were identical to those described for the training dataset in Section 2.1 “Training Dataset”.

Each WSI was analysed as an independent slide-level case, with no more than two slides derived from any individual needle biopsy case. The external test cohort consisted of 41 benign slides and 109 malignant slides. The malignant slides included 4 ISUP GG 1, 24 GG 2, 24 GG 3, 19 GG 4, and 38 GG 5 cases (Table 5). Although the slides were sourced through a single biobank, efforts were made to preserve dataset heterogeneity by including material from multiple contributing centres, retaining real-world staining variability, and limiting the number of slides contributed by any individual case. This approach was intended to reduce case-level redundancy while capturing a range of routine diagnostic conditions encountered in clinical practice.

Prior to inclusion in the study, all WSIs underwent quality assessment by two pathologists with more than 10 years of diagnostic experience and were deemed suitable for analysis. The external test dataset was entirely independent of the algorithm-development dataset, with no overlap between training and testing material. In addition, the inclusion of 41 benign slides enabled the assessment of true-negative classification and calculation of specificity.

Ground truth (GT) was established for all 150 slides by two GU pathologists. Both pathologists independently assessed the WSIs and were blinded to the AI outputs and the consensus interpretations of the participating pathologists. Any discrepancies were resolved through a consensus discussion. This consensus diagnosis served as the reference standard for all subsequent analyses.

**Figure 1:**
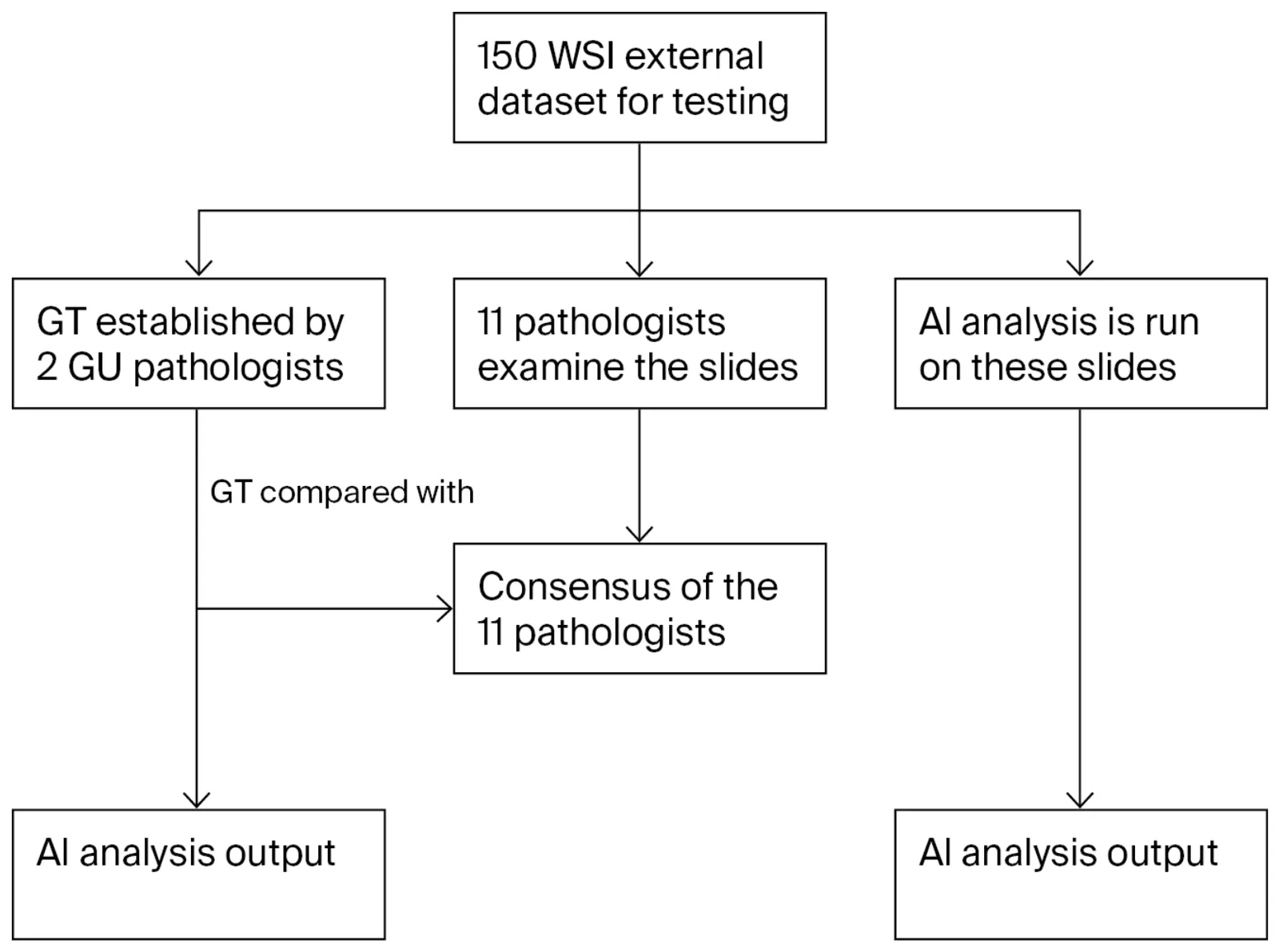
Study protocol showing establishment of ground truth, generation of 11-pathologist consensus, and parallel AI analysis of the external test dataset. AI outputs and pathologist consensus were compared with ground truth. Abbreviations: AI, artificial intelligence; GT, ground truth; GU, genitourinary; WSI, whole-slide image.

To provide a benchmark against routine diagnostic interpretation, each of the 150 WSIs was independently examined by 11 pathologists using digital pathology without AI assistance. The AI system analysed the same slides in parallel (Figure 1). For the pathologist arm of the study, a consensus diagnosis was determined using majority agreement for both benign-versus-malignant classification and ISUP GG assignment. Diagnostic performance comparisons were subsequently performed between the AI system and GT, and separately between the 11-pathologist consensus and GT.

IHC was not used during GT establishment. Consensus was achieved in all cases using H&E morphology alone, reflecting the diagnostic workflow under which the study was conducted. While this limits comparison with settings where ancillary testing is routinely available, it provides a pragmatic reference standard for evaluating AI-assisted screening under H&E-based diagnostic conditions. This aspect of the study may be particularly relevant to other groups developing pathology AI tools in similar healthcare settings.

All methods were performed in accordance with applicable ethical approvals, relevant guidelines, and institutional regulations.

### 2.4. Algorithm Training and Model Architecture

#### 2.4.1. Model Architecture

The AI system used a region-based convolutional neural network to classify and segment histopathological regions in haematoxylin and eosin (H&E)-stained prostate core needle biopsy whole-slide images (WSIs) [10]. Prostate biopsy assessment requires the detection, localisation, and separation of morphologically distinct components, including benign glands, stroma, inflammation, blood vessels, artefactual changes, and malignant glands showing different Gleason patterns. An instance-segmentation architecture was selected to provide region-level localisation rather than a single slide-level classification score.

The system was implemented using Mask R-CNN, which extends Faster R-CNN by adding a fully convolutional segmentation branch parallel to the classification and bounding-box regression branches. For each candidate region, the network identifies whether a relevant structure is present, assigns a histological class, refines its spatial coordinates, and generates a pixel-level mask. The design of the architecture is suited to prostate needle biopsies, in which tumour foci may be small, irregular, and intermingled with benign glands, stroma, inflammation, vessels, and artefacts. It also allows Gleason patterns 3, 4, and 5 to be localised separately when they coexist within a biopsy core. Because their relative distribution contributes to GG assignment, the resulting masks provide both grading-related spatial information and interpretable overlays.

ResNet-101 served as the feature-extraction backbone [11]. This 101-layer residual network uses identity shortcut, or skip, connections that bypass one or more convolutional layers. Residual connections facilitate gradient propagation during backpropagation, mitigate the vanishing-gradient problem, and enable the network to learn residual mappings rather than direct input–output transformations, thereby improving training stability and convergence. The resulting architectural advantages support the recognition of subtle morphological differences among benign mimics, low- and high-grade carcinomas, inflammatory aggregates, and artefactual tissue changes.

Feature extraction occurs hierarchically across successive ResNet stages, with increasing feature depth and decreasing spatial resolution. Earlier layers capture edges, colour variation, gland contours, nuclear density, and local texture, whereas deeper layers represent glandular architecture, stromal relationships, tumour growth patterns, and broader tissue organisation. The network can combine fine cytological detail with the larger-scale architectural information required for prostate biopsy interpretation.

A Feature Pyramid Network (FPN) was integrated with the ResNet-101 backbone to accommodate structures at different spatial scales [12]. Small malignant glands, lymphocytic aggregates, nerves, vessels, and focal artefacts occupy limited areas, whereas Gleason pattern 4 or 5 tumour may form larger expansile or poorly formed regions. The FPN combines feature maps from successive backbone levels through lateral 1 × 1 convolutions and element-wise addition with upsampled higher-level features. The top-down hierarchy enriches lower-level maps, which retain spatial detail, with semantic information from deeper layers, producing multi-scale representations that preserve both localisation accuracy and tissue context.

A fully convolutional Region Proposal Network (RPN) operates on the FPN feature maps to identify candidate tissue regions. Using predefined anchor boxes at multiple scales and aspect ratios, the RPN assigns each anchor an objectness score and a bounding-box regression offset. The objectness score estimates whether the anchor contains a relevant tissue component, while the offset refines its location. Candidate proposals are then passed to the classification and segmentation branches. Non-maximum suppression removes redundant overlapping proposals while retaining high-confidence regions, which is particularly useful when adjacent tiles or overlapping tissue structures generate duplicate detections.

RoI Align extracts a fixed-size feature representation for each candidate proposal. The classification head uses two fully connected layers to assign a categorical label, including benign tissue, Gleason pattern-associated tumour, and the contextual tissue classes used during model development. In parallel, bounding-box regression further refines the location of each proposal before segmentation.

The mask head operates alongside the classification and bounding-box branches. A sequence of convolutional layers followed by transposed convolution upsamples the RoI-aligned features to a fixed spatial resolution and produces a class-specific mask for each detected instance. Generating masks independently for each class separates mask prediction from classification and reduces inter-class competition. This is useful when adjacent tissue compartments share visual features and accurate boundaries are needed to distinguish benign glands, stroma, inflammatory aggregates, and malignant glands.

For each detected region, the final model output comprised a predicted tissue class, confidence score, bounding box, and pixel-level mask. The outputs were projected onto the WSI as colour-coded segmentation overlays, allowing pathologists to inspect the regions contributing to each prediction and the spatial distribution of predicted Gleason patterns.

#### 2.4.2. Training, Validation, and Internal Test Split

The dataset was partitioned into training, validation, and test sets using a 70:15:15 split, comprising 395 benign and 1083 malignant slides in the training set, 88 benign and 231 malignant slides in the validation set, and 86 benign and 232 malignant slides in the test set (Table 3).

**Table 3:** Dataset split.

	Train	Validation	Test	Total
Benign Slides	395	88	86	569
Malignant Slides	1083	231	232	1546

#### 2.4.3. Number and Distribution of Annotations by Histological Class

The annotation dataset included a broad range of histological classes to support tumour detection, Gleason pattern recognition, and discrimination from non-tumour tissue. Tumour-related annotations comprised 137,309 regions of Gleason pattern 3, 81,990 of Gleason pattern 4, and 44,012 of Gleason pattern 5. A further 140,775 benign tissue annotations represented the morphological spectrum of non-malignant prostate glands and surrounding benign tissue components.

Contextual classes included damaged tissue (6563 annotations), blood vessels (29,816), nerves (7266), perineural invasion (9920), inflammation (6839), and stroma (5087) (Table 4). These labels were included to help the model distinguish tumour from background structures and commonly encountered mimics. Inflammatory aggregates, damaged tissue, stromal fragments, vessels, and nerves may resemble or obscure tumour foci, particularly in small biopsy cores or slides affected by staining variation, tissue folding, or other artefacts. Their inclusion exposed the model to the wider tissue context encountered in routine diagnostic material.

The benign and Gleason pattern classes provided the principal training signal for tumour detection and GG-related pattern recognition. Representation was nevertheless uneven: damaged tissue, inflammation, stroma, and nerves were less extensively annotated than the benign and tumour classes, reflecting the practical constraints of model development in a resource-limited setting. This imbalance may reduce the model’s ability to recognise these non-tumour features consistently and may contribute to false-positive tumour predictions or grading errors.

Further development of the supervised model will require a larger and more diverse set of annotations for inflammatory aggregates, tissue artefacts, stromal changes, necrosis, nerves, blood vessels, and other benign mimics. Slides showing variable staining quality, tissue folding, crush artefact, inflammation, and low-volume tumour need to be included in the training data. Material from additional laboratories, scanners, and staining workflows will further improve generalisability, particularly in resource-constrained environments where pre-analytical variability is common and laboratory infrastructure may be less standardised.

**Table 4:** Number of annotations per histological class across all algorithm-development splits.

Class	Number of Annotations
Benign	140,775
Gleason pattern 3	137,309
Gleason pattern 4	81,990
Gleason pattern 5	44,012
Damaged Tissue	6563
Blood Vessels	29,816
Nerves	7266
Perineural Invasion	9920
Inflammation	6839
Stroma	5087

#### 2.4.4. Tile Selection Strategy

Each WSI was divided into smaller image tiles suitable for neural-network processing. A minimum tissue-content threshold was applied to exclude tiles composed predominantly of glass background or empty regions around the biopsy cores while retaining those containing sufficient tissue for analysis. Removing background-dominant tiles reduced the number of uninformative inputs, improved computational efficiency, and focused training on regions containing histological structures.

For supervised learning, tiles were selected based on their spatial overlap with ground-truth annotation masks generated by the pathologists. Tiles overlapping annotated regions were retained and assigned the corresponding histological class or classes. Eligible labels included benign tissue, Gleason pattern-associated tumour, inflammation, blood vessels, nerves, stroma, damaged tissue, and perineural invasion. This approach linked the training inputs directly to pathologist-defined regions of diagnostic relevance, allowing the model to learn directly from the pathologists.

Tiles that did not overlap with a ground-truth annotation mask were excluded because the class of non-annotated tissue could not be assigned reliably. Their omission reduced label uncertainty and avoided introducing ambiguous or potentially incorrect examples into the strongly supervised training set. This strategy balanced computational efficiency, annotation fidelity, and diagnostic relevance by retaining tissue-containing tiles with reliable class labels. However, it also reduced the model’s exposure to unannotated benign and contextual tissue patterns.

#### 2.4.5. Loss Function

A loss function is the model’s error score during training. The model makes a prediction, compares it with the pathologist’s annotation, and receives a numerical penalty for being wrong. Training repeatedly adjusts the model to reduce that penalty. For example, a pixel annotated as Gleason pattern 4 incurred a greater loss when the model assigned a higher probability to benign tissue or Gleason pattern 3. The loss decreased as confidence in the correct class increased. Cross-entropy quantified the disagreement between these predicted probabilities and the corresponding ground-truth label in the pathologist-derived annotation mask, applying larger penalties when the probability assigned to the correct class was low.

In the implementation used in this study, cross-entropy loss served as the training objective for the multiclass image-segmentation model. For each pixel or annotated region within a training tile, the model generated a probability distribution across the available classes, including background, benign tissue, Gleason patterns 3, 4, and 5, and the annotated contextual tissue categories.

Across the training dataset, this objective supported the learning of class-discriminative features needed to distinguish benign glands, different Gleason patterns, and non-tumour structures that may mimic or obscure malignant glands. The resulting class probabilities contributed to segmentation-mask generation, confidence estimation, and subsequent slide-level aggregation. They also enabled the model to represent greater uncertainty in heterogeneous, borderline, or artefactual regions.

As with other supervised learning methods, performance depended on the quality, consistency, and representativeness of the ground-truth annotations. The class imbalance could also influence learning because some contextual categories were less frequently represented than benign tissue and Gleason pattern annotations.

#### 2.4.6. Post-Processing

Post-processing refers to the computational steps applied after the AI model has generated its initial tile-level predictions. In this study, these steps combined and spatially aligned the predicted masks, resolved overlapping predictions from adjacent tiles, and mapped the results back to their correct locations on the original whole-slide image. The strategy produced a coherent slide-level output that is suitable for visualisation and quantitative analysis.

Because gigapixel-resolution whole-slide images (WSIs) exceeded the available hardware memory for native-resolution aggregation, post-processing was performed on spatially downsampled representations of the full-resolution segmentation outputs. Simultaneous storage and processing of multiple masks, class probabilities, confidence scores, and overlapping tile predictions would otherwise require substantial computational resources. The pixel coordinates of each instance mask were scaled by a fixed downsampling factor before WSI-level aggregation, reducing memory requirements while preserving the relative positions, shapes, and spatial relationships of the detected regions. The downsampled masks could then be reconstructed efficiently across WSIs of varying sizes.

Each WSI was divided into image tiles according to a predefined tessellation pattern and stride. During post-processing, the predicted mask from each tile was mapped to its original position in the global slide coordinate system using the same stride information, producing a spatially aligned WSI-level composite mask. Because adjacent tiles could overlap, the same tissue region could contribute more than one prediction. At these locations, per-pixel class scores were aggregated across all contributing predictions rather than relying on a single tile. This reduced tile-boundary artefacts and discontinuities and improved the spatial consistency of the reconstructed output.

After assembly of the WSI-level mask, the inverse downsampling transformation was applied to project the instance coordinates back into the original full-resolution slide coordinate space. This restored correspondence with the native WSI and allowed the final masks to be overlaid accurately for visualisation, review, and downstream analysis.

The resulting slide-level outputs comprised predicted tissue masks, class labels, bounding regions, and associated confidence scores. These were serialised in a structured data format to support slide-level aggregation, quantitative analysis, visual-overlay generation, and integration with digital pathology viewing platforms. The structured outputs also enabled pathologists to examine the spatial distribution of predicted benign tissue, Gleason pattern-associated tumour regions, and contextual tissue classes.

Overall, the workflow balanced computational feasibility with spatial accuracy by performing aggregation on downsampled masks and restoring the results to the native coordinate system. This approach retained clinically relevant spatial information while reducing the memory required to process large prostate biopsy WSIs, an important consideration in resource-constrained development and deployment settings.

#### 2.4.7. Slide-Level Aggregation

Slide-level aggregation converted the multiple tissue instances detected within each whole-slide image (WSI) into a compact feature representation for binary classification. Instance-level attributes were extracted from the structured segmentation outputs and combined to determine whether the overall slide was more consistent with benign tissue or malignancy.

For each detected instance, three primary features were calculated: the predicted class confidence score, segmented instance area, and a class-specific instance weighting factor. The confidence score represented the estimated probability that an instance belonged to benign tissue, Gleason pattern 3, Gleason pattern 4, Gleason pattern 5, or one of the contextual tissue categories; lower scores indicated greater uncertainty. The segmented area was derived from the corresponding pixel-level mask and represented the spatial extent of the predicted tissue component. This allowed the classifier to distinguish small isolated predictions from larger or more extensive tumour regions. Although a small predicted tumour focus may contribute less to the slide-level prediction than a larger contiguous area, it remained relevant in a screening-oriented workflow designed to minimise missed malignancies.

The class-specific weighting factor reflected the relative diagnostic importance of each predicted tissue class. While contextual classes contribute to accuracy, tumour-associated classes, particularly Gleason patterns 4 and 5, could carry greater weight due to their diagnostic significance.

These attributes were aggregated across the WSI to create slide-level feature vectors summarising the distribution, confidence, and extent of the predicted tissue classes. This allowed the downstream classifier to integrate information from multiple regions rather than rely on a single prediction. Such integration is relevant in prostate needle biopsies, where tumour may be focal, multifocal, or heterogeneously distributed and may occur near benign mimics, inflammatory aggregates, or artefacts.

Binary slide classification was performed using a gradient-boosted decision tree classifier trained on the extracted feature vectors. This method can model non-linear relationships and interactions among structured variables, including confidence scores, predicted tissue area, and class-specific weights. The classifier used these aggregated segmentation-derived features to assign each WSI a benign or malignant prediction.

This two-stage framework separated pixel- or instance-level tissue recognition from slide-level classification. The segmentation model first identified and localised histological regions of interest, after which the gradient-boosted classifier integrated the outputs into a clinically interpretable screening result. These predictions required pathologist interpretation and were not used as final diagnoses.

## 3. Results

### 3.1. Run Time and Hardware

The AI system generated segmentation outputs within approximately 45 s to 1 min per whole-slide image (WSI). Assuming one WSI per biopsy core and sequential processing, a typical case comprising 12–18 cores would require approximately 12–18 min. This extrapolation indicates computational feasibility under the study conditions but must not be interpreted as a formal measure of real-world reporting or turnaround time.

**Figure 2:**
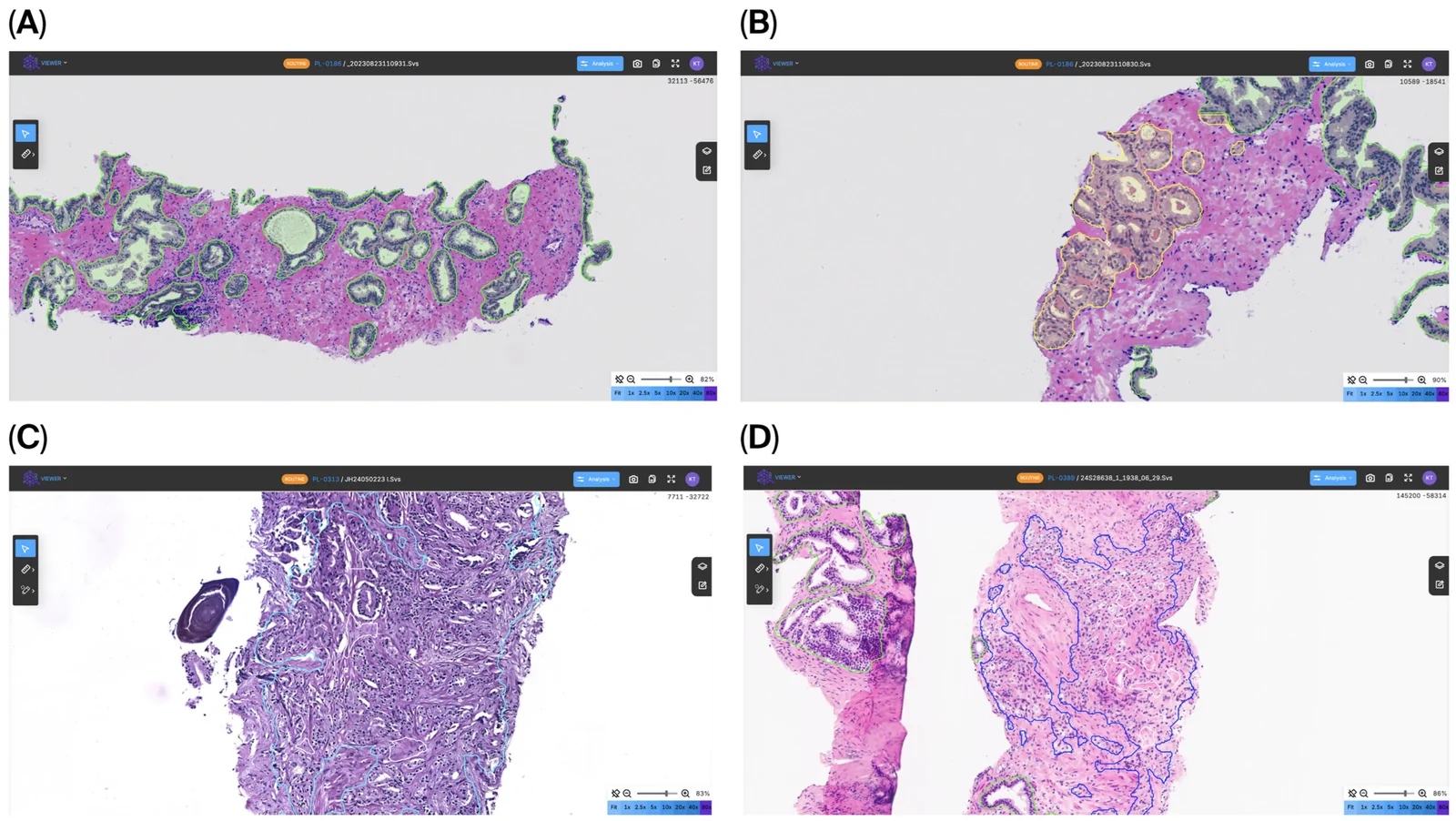
Representative segmentation outputs generated by the artificial intelligence system. Colour code: green: benign tissue; yellow: Gleason pattern 3; light blue: Gleason pattern 4; deep blue: Gleason pattern 5. Abbreviation: AI, artificial intelligence. (**A**) Benign prostatic tissue segmented in green; (**B**) regions predicted as Gleason pattern 3 segmented in yellow, with adjacent benign tissue segmented in green; (**C**) regions predicted as Gleason pattern 4 segmented in light blue; (**D**) regions predicted as Gleason pattern 5 segmented in deep blue, with adjacent benign tissue segmented in green. Abbreviation: AI, artificial intelligence.

The outputs were displayed as colour-coded segmentation overlays to support interpretability, with benign tissue shown in green, Gleason pattern 3 in yellow, Gleason pattern 4 in light blue, and Gleason pattern 5 in deep blue (Figure 2). These overlays allowed pathologists to examine the spatial distribution of predicted tissue classes, identify regions contributing to the AI-assigned grade, and assess whether the predictions corresponded to plausible morphology.

Inference was performed using an Amazon Web Services Elastic Compute Cloud EC2 g4dn.2xlarge instance equipped with one NVIDIA T4 Tensor Core GPU with 16 GB GPU memory, 8 virtual CPUs, and 32 GiB system RAM. This cloud-based configuration provided sufficient capacity for WSI-level inference without requiring dedicated high-performance hardware in the local laboratory. Such an approach may be scalable across resource-limited or geographically distributed centres, provided that data governance, privacy, network bandwidth, cost, and regulatory requirements are appropriately addressed.

The observed processing time supports further evaluation of the system in either a pre-analysis or second-read workflow. Segmentation outputs could be generated before a pathologist examines the data to highlight suspicious regions or applied after the initial assessment as a safety check for tumour foci requiring re-examination. However, the study did not assess total case turnaround time, pathologist reporting or interaction time, time saved during slide reporting, or the learning period required to use the system. The estimated case-level processing time also excluded slide upload, image transfer, preprocessing, quality control, case loading, viewer integration, and pathologist review of the AI outputs.

### 3.2. ISUP Grade Group Concordance

Agreement between AI-predicted and ground truth (GT) ISUP GG was further assessed using Cohen’s kappa statistics. In addition to unweighted Cohen’s kappa, linearly weighted and quadratically weighted kappa values were calculated to account for the ordinal nature of ISUP GG classification. Grading disagreements in prostate cancer are not all equivalent; a discrepancy between adjacent GGs has different clinical and statistical implications from a discrepancy across widely separated categories. The full confusion matrix comparing GT and AI-predicted ISUP GGs is shown in Table 5.

Agreement between GT and AI varied according to the kappa metric applied. The unweighted Cohen’s kappa was 0.53 (95% CI: 0.44–0.615), indicating moderate agreement when all grading discrepancies were treated equally. However, agreement improved substantially when the ordinal structure of ISUP GGs was considered. The linearly weighted Cohen’s kappa was 0.76 (95% CI: 0.68–0.82), reflecting substantial agreement, while the quadratically weighted Cohen’s kappa increased further to 0.87 (95% CI: 0.79–0.92), indicating high ordinal agreement when the magnitude of grading differences was considered. This progressive increase from unweighted to weighted kappa suggests that many AI–GT discrepancies were limited to adjacent or near-adjacent GGs rather than representing major grading discordance.

ISUP GGs represent an ordered grading system. In such systems, a one-grade discrepancy, for example, between GG 2 and GG 3, is statistically and clinically different from a wider discrepancy, such as between GG 2 and GG 5. Therefore, while exact categorical agreement between GT and AI was moderate, the weighted kappa results indicate stronger ordinal concordance when the magnitude of grading differences was taken into account (Table 6).

Among the 109 malignant cases, exact concordance between AI-predicted and GT ISUP GG was observed in 57 cases (52.3%). When discordance occurred, the AI more frequently assigned a higher GG than the GT. Specifically, the AI assigned a higher GG in 47 cases and a lower GG in five cases. Most disagreements were limited to one GG, while fewer cases showed discrepancies of two or more GGs (Table 5).

Several grading discrepancies warrant closer consideration. None of the four cases assigned ISUP GG 1 in the GT were classified as GG 1 by the AI; instead, two were classified as GG 2 and two as GG 3. Among the 24 GT GG 2 cases, the AI classified 16 as GG 3, one as GG 4, and two as GG 5. For cases designated as GG 3 in the GT, eight were classified as GG 5 by the AI, while three were downgraded to GG 2. In contrast, agreement was stronger among higher-grade tumours. Among GT GG 4 and GG 5 cases, only one GG 5 case was classified as GG 3, representing a discrepancy of more than one GG.

Although most discordant cases differed by only one GG, the larger deviations remain important and require careful interpretation. The predominant causes of misclassification were histological and technical artefacts, including tissue folding, suboptimal staining, staining artefacts, scanning artefacts, air bubbles, and dense inflammation. These features were occasionally interpreted by the AI as tumour or as a higher Gleason pattern. For example, two cases diagnosed as GG 2 by GU pathologists during GT establishment were classified by the AI as GG 5 because tissue artefacts were interpreted as Gleason pattern 5 tumour. In another GG 2 case, dense inflammation was misclassified as Gleason pattern 5, contributing to an AI-assigned grade of GG 4. This reflects an important error pattern in which inflammatory aggregates may be misinterpreted as high-grade tumour.

Among the 41 GT benign cases, three were incorrectly classified as malignant by the AI. Two benign slides were classified as GG 5 because artefacts were interpreted as high-grade tumour; one showed tissue folding, while the other showed staining artefact. The remaining benign slide was classified as GG 2, with folded tissue erroneously identified as tumour. These findings indicate that although the AI system showed high sensitivity for tumour detection, false-positive predictions may arise in the presence of technical artefacts or benign mimics.

More subtle grading errors were also observed. Of the 24 cases classified as GG 2 in the GT, 16 were classified as GG 3 by the AI. In seven of these cases, small areas of Gleason pattern 3 tumour were misclassified as Gleason pattern 4, resulting in an upgrade in GG. Many of these slides had limited tumour burden, where even a small number of misclassified regions was sufficient to shift the overall AI-assigned classification from GG 2 to GG 3. In the remaining cases, staining artefacts, tissue folding, and air bubbles were interpreted as Gleason pattern 4 tumour, thereby influencing the AI output.

The AI demonstrated meaningful ordinal agreement with GT but also showed a tendency toward overgrading, particularly in cases affected by artefacts, inflammation, staining variability, or limited tumour volume. These findings argue against using the AI grade without morphological assessment. AI-generated grading outputs may help highlight suspicious regions, but they may also introduce interpretive bias if accepted uncritically.

### 3.3. Benign Versus Malignant Classification Performance

The diagnostic performance of the AI system and the consensus interpretation of 11 pathologists was evaluated against ground truth (GT) established by two expert GU pathologists. Binary benign-versus-malignant performance metrics were calculated separately for the AI system and the pathologist consensus.

Among the 109 GT malignant cases, the AI correctly identified all malignant slides, achieving a sensitivity of 100.0% (109/109), with no false-negative predictions. In comparison, the pathologist consensus correctly identified 107 of 109 malignant slides, corresponding to a sensitivity of 98.2%. The two missed cases contained small foci of low-grade Gleason pattern 3 carcinoma, indicating that the AI detected two low-volume tumour foci not identified by the unaided pathologist consensus.

Among the 41 GT benign cases, the AI correctly classified 38 slides, corresponding to a specificity of 92.7% (38/41), with three false-positive predictions. The pathologist consensus correctly classified 39 of 41 benign slides, corresponding to a specificity of 95.1%, with two false-positive interpretations. Thus, the pathologist consensus showed slightly greater specificity, whereas the AI showed higher sensitivity. This modest difference may reflect the sensitivity–specificity trade-off commonly encountered in screening-oriented systems.

**Figure 3:**
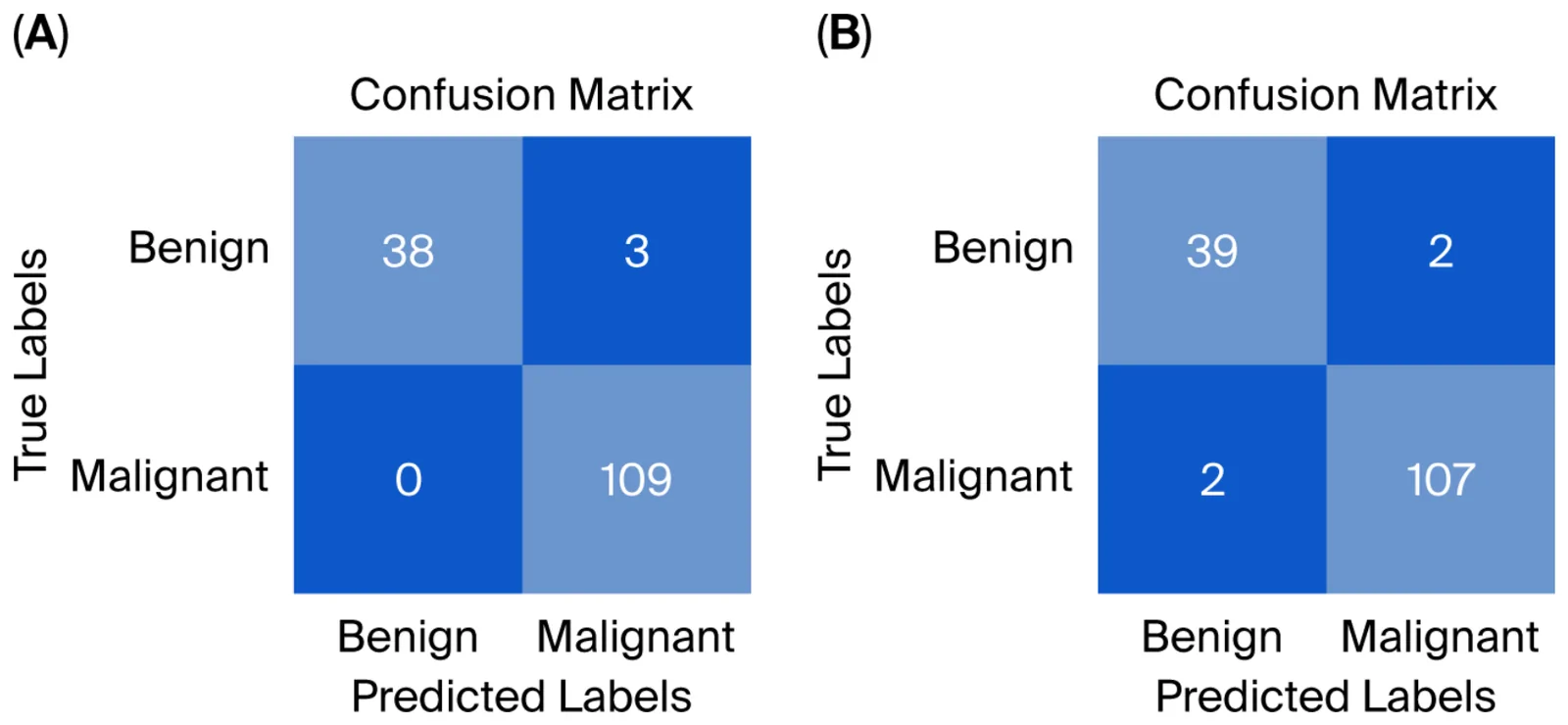
(**A**) “GT vs. AI” confusion matrix for benign-versus-malignant classification. The AI correctly classified 38/41 benign slides and 109/109 malignant slides, with three false-positive and no false-negative predictions. (**B**) “GT vs. 11-pathologist consensus” confusion matrix for benign-versus-malignant classification. The 11-pathologist consensus correctly classified 39/41 benign slides and 107/109 malignant slides, with two false-positive and two false-negative interpretations. Cell values indicate the number of slides. Abbreviations: AI, artificial intelligence; GT, ground truth.

Overall diagnostic accuracy was comparable between the two approaches. The AI achieved an accuracy of 98.0%, while the pathologist consensus achieved an accuracy of 97.3%. Against GT, the AI correctly classified 147 of 150 slides, compared to 146 of 150 for the pathologist consensus. The AI showed performance comparable to the 11-pathologist consensus for binary tumour detection. The corresponding confusion matrices are shown in Figure 3.

A detailed performance breakdown is provided in Table 7. Against GT, the AI achieved a precision, or positive predictive value (PPV), of 97.3% and a recall, or sensitivity, of 100.0%. The resulting F1 score was 98.6%. The negative predictive value (NPV) was 100.0%, as all slides classified as benign by the AI were benign according to GT. Specificity was 92.7%, reflecting the three false-positive predictions. Cohen’s kappa was 0.95, indicating near-perfect agreement with GT.

The pathologist consensus achieved an accuracy of 97.3%, with a PPV of 98.2% and a sensitivity of 98.2%. The F1 score was 98.2%, while the NPV and specificity were both 95.1%. Cohen’s kappa was 0.93, also indicating near-perfect agreement with GT. Both approaches performed strongly in binary tumour detection, with slightly different performance profiles.

False-positive and grading errors were primarily associated with histological and technical artefacts. Among the benign cases misclassified by the AI, two were assigned ISUP GG 5 because tissue folding and staining artefact were interpreted as high-grade tumour. A third benign case was assigned GG 2 because folded tissue was incorrectly identified as tumour. Dense inflammatory aggregates were also occasionally misclassified as higher-grade tumour. These findings identify artefacts, inflammation, and benign mimics as important sources of error and reinforce the need for pathologist supervision.

The annotation strategy may also have contributed to false-positive predictions. High-grade prostatic intraepithelial neoplasia and atypical small acinar proliferation were annotated as Gleason pattern 3 during model development to prioritise sensitivity and highlight potentially suspicious foci. While consistent with the model’s intended role as an assistive screening tool, this approach may increase false-positive tumour detection in cases with borderline atypical glands or limited suspicious foci. In the present dataset, two slides contained focal high-grade prostatic intraepithelial neoplasia and one contained atypical small acinar proliferation; however, all three also showed extensive invasive carcinoma, and these focal findings did not affect the overall grade assignment.

The absence of false-negative AI predictions is encouraging for a screening-oriented application, given the clinical consequences of missed malignancy. However, the false-positive predictions and grading errors emphasise the need for human oversight. AI outputs may direct attention to suspicious regions but could introduce interpretive bias if accepted without critical examination.

The AI system and pathologist consensus demonstrated strong and complementary performance. The AI achieved higher sensitivity with no false-negative predictions, while the pathologist consensus showed slightly higher specificity and fewer false-positive classifications.

**Table 7:** Comparison of AI performance and 11-pathologist consensus performance against ground truth for benign versus malignant classification. Abbreviations: AI, artificial intelligence; CI, confidence interval; GT, ground truth; NPV, negative predictive value; PPV, positive predictive value. Values are percentages with 95% CIs.

Metric	GT vs. AI	GT vs. 11-Pathologist Consensus
Accuracy	98.0% (95.3–100.0%)	97.3% (94.0–100.0%)
Precision (PPV)	97.3% (93.7–100.0%)	98.2% (94.5–100.0%)
Recall (Sensitivity)	100.0% (100.0–100.0%)	98.2% (94.5–100.0%)
F1 Score	98.6% (95.2–100.0%)	98.2% (94.5–100.0%)
NPV	100.0% (100.0–100.0%)	95.1% (87.8–100.0%)
Specificity	92.7% (82.9–100.0%)	95.1% (87.8–100.0%)
Cohen Kappa	0.95 (0.88–1.00)	0.93 (0.85–0.99)

### 3.4. Statistical Analyses

All statistical analyses were performed using Python, version 3.8, together with established open-source scientific computing libraries. Descriptive statistics were used to summarise the composition of the dataset and the distribution of diagnostic categories. These included frequency distributions for benign and malignant cases, ISUP GG categories, and AI-predicted versus ground truth classifications. Where applicable, numerical variables were summarised using means and standard deviations. Descriptive analyses were performed using NumPy, version 1.23.5, and Pandas, version 1.5.3.

Classification performance was evaluated by comparing AI outputs and pathologist consensus diagnoses against the ground truth established by expert GU pathologists. For benign versus malignant classification, standard diagnostic performance metrics were calculated, including sensitivity, specificity, positive predictive value (PPV), negative predictive value (NPV), F1 score, and overall accuracy. Sensitivity was used to quantify the proportion of malignant cases correctly identified, while specificity measured the proportion of benign cases correctly classified. PPV represented the proportion of positive predictions that were truly malignant, whereas NPV represented the proportion of negative predictions that were truly benign. The F1 score was calculated as a combined measure of precision and sensitivity, providing a summary measure of classification performance when balancing false-positive and false-negative predictions. Overall accuracy represented the proportion of all correctly classified cases.

These classification metrics were computed using the Scikit-learn library, version 1.2.2. Confusion matrices were generated to summarise true-positive, true-negative, false-positive, and false-negative classifications for both the AI system and the pathologist consensus. These matrices provided the basis for calculating the diagnostic performance metrics and allowed direct comparison between AI outputs, pathologist consensus, and ground truth.

Agreement between predicted and reference classifications was assessed using Cohen’s kappa statistics. For binary benign versus malignant classification, Cohen’s kappa was used to measure agreement beyond chance between the AI system and ground truth, and between pathologist consensus and ground truth. For ISUP GG assignment, kappa statistics were used to assess grading concordance between AI-predicted GGs and ground truth GGs. Because ISUP GGs represent an ordinal classification system, weighted kappa analyses were also performed. Both linearly weighted and quadratically weighted Cohen’s kappa values were calculated to account for the magnitude of grading disagreement, recognising that discrepancies between adjacent GGs are less severe than discrepancies across widely separated GGs.

Cohen’s kappa coefficients were calculated using functions from the Scikit-learn metrics module. Confusion matrices were constructed using Scikit-learn and visualised using Matplotlib, version 3.6. Confidence intervals were calculated for the reported diagnostic performance metrics and agreement statistics to provide an estimate of statistical uncertainty. All analyses were conducted at the slide level, with each whole-slide image treated as an independent unit of analysis.

## 4. Discussion

This study is among the relatively few investigations to compare an AI system directly with the consensus interpretation of multiple pathologists using a ground truth (GT) established by GU pathologists. It also contributes to the limited but growing evidence on the potential role of AI-assisted approaches in histological tumour grading. Because the evaluation was retrospective and conducted under controlled conditions, the findings can only be interpreted as evidence of the system’s potential utility as an assistive screening tool rather than readiness for autonomous clinical deployment.

For binary classification of benign and malignant tissue, the AI system demonstrated strong tumour-detection performance relative to the pathologist consensus. A study published in 2021 similarly reported that an AI system outperformed 10 of 14 participating pathologists in prostate cancer detection [13], while several studies and reviews have discussed the potential for AI to contribute constructively to pathology workflows [14,15,16]. Nevertheless, like the present study, the 2021 investigation was conducted in a controlled research environment; validation in real-world clinical settings is required before clinical impact can be established [13,17]. The data from this study shows that both the AI system and the GU pathologists correctly identified tumour in the two cases missed by the broader group of pathologists, consistent with the established importance of subspecialty expertise in prostate biopsy assessment [18]. However, access to such expertise is not uniform across healthcare settings and may be particularly limited in developing countries, where diagnostic services are also affected by the global shortage of pathologists [19].

A related challenge in routine histopathological reporting is the volume of tissue that can be examined within a limited timeframe. Examining a greater volume of tissue may increase the likelihood of detecting small or inconspicuous tumour foci. This principle underlies the use of saturation prostate biopsies in selected clinical scenarios, in which larger tissue volumes are sampled to exclude malignancy more confidently [20]. However, increased sampling also generates additional slides or tissue levels for assessment, adding to the workload of already constrained pathology services. In principle, AI-assisted screening could support the analysis of larger tissue volumes by highlighting suspicious regions, helping pathologists prioritise their assessment, and facilitating the evaluation of deeper sections that might not otherwise be examined routinely [21]. The present findings support further investigation of the system as a second-read or safety-check mechanism. Its practical value, however, must be established through prospective, workflow-based validation. This interpretation is consistent with the emerging view that AI is best used to augment rather than replace pathology practice [22,23].

In the centrally sourced, external slide-level evaluation set, the AI system achieved performance comparable to the pathologist consensus for benign-versus-malignant classification. The balance between sensitivity and specificity is particularly important for a screening application because excessive false-positive detections could increase workload, reduce user confidence, and undermine the practical value of the system. The AI demonstrated slightly lower specificity than the pathologist consensus, reflecting a sensitivity–specificity trade-off that must be carefully evaluated before clinical implementation.

For ISUP GG assignment, the AI system tended to overgrade cases relative to both the GT and the pathologist consensus. This tendency resulted from a combination of genuine model errors and challenging histological or technical features. Small tumour foci, inflammatory aggregates, crushed or folded tissue, staining variation, and scanning artefacts were occasionally interpreted as higher Gleason patterns. Image quality and suboptimal staining have previously been associated with similar errors [24]. The findings emphasise the importance of high-quality tissue processing, staining, and slide scanning, as pre-analytical variability can substantially influence AI performance [25].

In a screening context, overgrading may be more readily identified and corrected by a pathologist than a false-negative result. Nevertheless, overgrading remains clinically important. If accepted without appropriate scrutiny, an erroneously high GG could affect risk stratification, treatment planning, patient counselling, and eligibility for active surveillance. Consequently, AI-predicted higher-grade regions require careful pathologist adjudication, particularly when they occur in areas affected by artefact or inflammation, contain limited tumour, or are discordant with the surrounding morphology.

The need for laboratory-specific calibration or model adaptation should be assessed locally. In this context, calibration refers to training or fine-tuning the AI system using locally generated data to address systematic effects associated with staining protocols, scanners, tissue-preparation methods, laboratory equipment, and diagnostic workflows. Although many AI models can generalise across datasets, targeted calibration may be beneficial when performance falls below predefined acceptance thresholds [26]. For example, calibration may help reduce excessive false-positive tumour detections that would otherwise compromise the value of a screening system. Even minor changes to local processes, protocols, or equipment can affect model performance. This needs to trigger local validation and, where necessary, recalibration. Relevant strategies are discussed further in Section 6, “Calibration strategies to reduce overgrading from artefacts and inflammation”.

The dataset was generated in a developing-country setting, where digital pathology infrastructure, automated staining systems, standardised laboratory workflows, subspecialist availability, and large-scale annotation resources may be more limited than in highly resourced centres. Manual H&E staining introduced real-world variation in staining intensity, contrast, and colour balance. Access to immunohistochemistry was also limited, and immunohistochemical findings were not incorporated into GT development, reflecting the practical diagnostic environment in which the study was conducted. All slides were scanned using a single scanner, which improved consistency but prevented assessment of scanner-level generalisability. Cloud-based computing provided a practical means of running AI inference without substantial on-site computational infrastructure. These features position the study as a resource-aware AI development and validation experience that may inform groups developing pathology AI systems under similar constraints.

As artificial intelligence is increasingly incorporated into diagnostic decision-making, it is important to recognise that different types of error do not carry equivalent clinical consequences. Failure to detect prostate malignancy, for example, could delay treatment and adversely affect patient outcomes. The clinical evaluation of AI systems should consider measures beyond overall accuracy [27]. Against this background, the absence of false-negative malignant classifications in the present study supports further evaluation of the system as an assistive screening tool in which sensitivity is a central performance consideration.

A principled learning framework should incorporate uncertainty quantification, enabling the model to indicate the confidence associated with each prediction. Explicit deferral strategies could then direct cases or regions associated with substantial uncertainty to expert human review. Model optimisation should be guided by clinically meaningful objectives, including minimising false-negative results in high-risk scenarios, reducing clinically significant grading errors, and aligning AI outputs with real-world diagnostic thresholds. Collectively, these measures are necessary to ensure that AI systems are not only accurate in retrospective datasets but also safe, reliable, interpretable, and aligned with the priorities of clinical care [28].

## 5. Comparison to Other Studies

A comparison with previously published studies places the present model within the evolving field of AI-based prostate cancer detection on whole-slide images (WSIs). Several recent studies have demonstrated that AI systems can achieve high diagnostic performance for tumour detection in prostate biopsy specimens, although reported sensitivity and specificity vary according to dataset size, case composition, slide quality, scanner type, staining variability, model architecture, and the intended clinical use of the algorithm. Therefore, direct comparison across studies should be interpreted cautiously, as differences in study design and validation strategy may influence reported performance.

Raciti P. et al. (2023) [29] reported a sensitivity of 97.4% and specificity of 94.8% on a dataset of 610 WSIs. Santa–Rosario J.C. et al. (2024) [30] demonstrated a balanced diagnostic profile, with sensitivity and specificity of 96.6% and 96.7%, respectively, using a larger cohort of 1279 WSIs. In contrast, Pantanowitz L. et al. (2020) [31] reported a lower sensitivity of 90.1% but a notably high specificity of 99.6% across 2501 WSIs (Table 8). These studies illustrate the inherent trade-off between sensitivity and specificity in AI-based tumour detection. Models optimised to minimise false-negative results may show slightly lower specificity, while models optimised to reduce false-positive findings may show comparatively lower sensitivity.

The present study achieved a sensitivity of 100% and a specificity of 92.7% on an external evaluation dataset of 150 WSIs. Despite this dataset being smaller than those used in some previously published studies, it was curated to preserve a degree of heterogeneity by limiting inclusion to no more than two slides per case and sourcing material from multiple participating institutions. This approach was intended to reduce case-level redundancy and capture inter-institutional variability in tissue preparation and H&E staining. In addition, the slides were generated under real-world resource-constrained conditions, including manual staining workflows and limited access to automated laboratory infrastructure. These factors may have introduced pre-analytical variability but also make the findings relevant to settings where digital pathology and AI implementation are emerging.

The model’s ability to identify all tumour-positive slides in the present dataset is noteworthy from a screening perspective, as false-negative predictions are of particular concern in prostate biopsy assessment. A missed malignant focus could delay diagnosis, defer appropriate treatment, or falsely reassure clinicians and patients. The absence of false-negative results in this retrospective evaluation supports further assessment of the model as an assistive screening tool.

The specificity of 92.7%, corresponding to three false-positive cases, was lower than that reported in some larger studies. These false-positive predictions were mainly associated with artefacts and benign mimics, including tissue folding and staining-related artefacts. This reduction in specificity reflects a clinically relevant trade-off in a screening-oriented model that prioritises sensitivity. In an assistive workflow, false-positive AI outputs may be revisited and corrected by a pathologist, whereas false-negative outputs carry a greater risk of missed malignancy. Nevertheless, excessive false-positive detections could increase workload, reduce confidence in the system, and limit practical adoption.

Importantly, the present study differs from several larger validation studies by explicitly evaluating an AI model developed under resource-aware conditions. All slides were scanned using a single scanner, which provided consistency but limits assessment of scanner-level generalisability. At the same time, inference was performed using cloud-based computing, suggesting a potentially practical route for AI deployment in geographically distributed or infrastructure-limited centres. These features provide useful context for groups developing pathology AI systems under similar constraints.

**Table 8:** Comparison of similar studies with data from the present study where AI was used to identify tumour on needle biopsies. Abbreviations: AI, artificial intelligence; WSI, whole-slide image.

Study Name	Year of Study	Sensitivity (%)	Specificity (%)	Dataset Size (WSIs)
Raciti P et al. [29]	2023	97.4	94.8	610
Santa–Rosario JC et al. [30]	2024	96.6	96.7	1279
Pantanowitz L et al. [31]	2020	90.1	99.6	2501
Saraf SA et al. (Present study)	2026	100	92.7	150

## 6. Calibration Strategies to Reduce Overgrading from Artefacts and Inflammation

The present study showed that the AI system tended to overgrade a subset of prostate needle biopsy slides, particularly when benign or low-grade areas contained features that partially mimicked higher-grade carcinoma. These errors appeared to arise from both histological and technical factors. Histological contributors included dense inflammatory aggregates, lymphoid follicles, stromal condensation, tangentially sectioned glands, atrophic glands, crushed glands, high-grade prostatic intraepithelial neoplasia, atypical small acinar proliferation, and fragmented benign glands at tissue edges. Technical contributors included tissue folds, staining variability, overstaining, understaining, air bubbles, focus variation, scanning artefacts, and damaged tissue. In these settings, the model may generate small false-positive or disproportionately high-grade segmentation masks, which can influence the final slide-level ISUP GG. This is particularly relevant in prostate biopsy grading because even a small predicted component of Gleason pattern 4 or pattern 5 may shift the AI-assigned GG upward, especially in otherwise low-volume Gleason pattern 3 disease.

Calibration must focus on the specific error patterns observed in this study, rather than simply increasing the size of the training set. Cases in which benign tissue, inflammation, artefact, or Gleason pattern 3 was overcalled as higher-grade tumour would need to be identified systematically and reintroduced into training as hard-negative or borderline examples. These cases must include benign mimics, inflammatory foci, crushed or folded tissue, small atypical glands, tangentially sectioned glands, stromal fragments, and technically suboptimal areas. Importantly, these regions require explicit annotation rather than being treated as background. Additional contextual classes, such as lymphoid aggregates, tissue folds, air bubbles, damaged tissue, overstained or understained tissue, crushed tissue, and benign glandular mimics, may help the model distinguish true high-grade tumour from non-neoplastic or artefactual tissue. This is important because many false-positive high-grade predictions arise in visually complex regions rather than in simple background areas.

Hard-negative mining will also be useful in subsequent model iterations. After each round of model development, discordant cases could be identified to isolate recurrent sources of overgrading. Regions falsely labelled by the AI as Gleason pattern 4 or 5 can then be selectively sampled more frequently during retraining. This will allow the model to learn directly from its most clinically relevant errors. In parallel, the training dataset requires enrichment with genuine examples of low-grade carcinoma, small-volume Gleason pattern 3, borderline Gleason pattern 3 versus 4 morphology, and benign mimics resembling fused, poorly formed, or cribriform glands. This will address not only artefact-related errors, but also potential under-representation of the morphological spectrum of low-grade and borderline lesions.

Loss-function optimisation can provide another route to reducing overgrading. If high-grade tumour classes are overemphasised during training, or if small high-grade predictions have excessive influence on the final slide-level output, the model may become overly sensitive to limited suspicious regions. Class weighting, focal loss, or targeted sampling strategies can be used to improve recognition of under-represented non-tumour classes, including inflammation, damaged tissue, artefact, and stroma. The aim would not be to reduce sensitivity for clinically significant carcinoma, but to improve the distinction between true high-grade cancer and artefactual or inflammatory mimics. Any such adjustment needs to be tested carefully using a validation set enriched for both true high-grade cancer and difficult benign or low-grade cases.

At the prediction stage, confidence-threshold tuning and probability calibration may help reduce inappropriate high-grade calls. For example, validation-set-based threshold adjustment could require a higher confidence score or a minimum area threshold before a small predicted Gleason pattern 4 or 5 region is allowed to contribute to the final GG assignment. Probability calibration methods, such as temperature scaling, will also help align the model’s confidence with the likelihood that a prediction is correct. This will be particularly useful when high-confidence predictions occur in artefact-prone regions. A calibrated model will need to distinguish a genuine tumour from an isolated false-positive prediction caused by poor tissue quality or benign changes.

Post-processing rules may provide an additional safeguard against clinically meaningful overgrading. Isolated small foci of predicted Gleason pattern 4 or 5 could be down-weighted if they fall below a predefined area threshold, lack spatial continuity, or overlap with regions classified as artefact, inflammation, damaged tissue, or tissue fold. Similarly, slide-level ISUP GG assignment could incorporate both the amount and distribution of predicted high-grade tumour, rather than relying only on the presence of any high-grade mask. A small isolated high-grade prediction in an artefactual area could be flagged for mandatory pathologist review instead of automatically altering the final AI-assigned grade. Such rules may help reduce benign-to-malignant misclassification and inappropriate upgrading of GG 1 or GG 2 cases.

Quality-control modules could also be incorporated before diagnostic inference. A pre-analytic QC algorithm can identify slides or tissue regions with poor focus, folds, staining irregularity, bubbles, pen marks, or other artefacts. These regions could then be excluded from grading, assigned lower confidence, or highlighted for pathologist review. This would separate two related but distinct tasks: determining whether the image is technically suitable for grading and assigning a tumour grade only to interpretable tissue. Such an approach can be particularly useful in resource-limited settings, where staining and slide preparation can show greater variability.

Finally, the calibration process requires assessment of stain and scanner robustness. Although colour augmentation was used during development, subsequent versions need to incorporate stain normalisation, broader colour augmentation, scanner-specific calibration, and validation on images generated using different scanners and staining workflows. This would help determine whether overgrading is influenced by scanner optics, colour profile, sharpness, compression, or staining characteristics. A multi-scanner, multi-institutional calibration set would be particularly useful for identifying systematic acquisition-related errors. Discordant AI outputs also warrant analysis through a structured feedback loop, allowing the model to be refined while preserving high sensitivity for tumour detection.

These calibration strategies are intended to preserve the model’s tumour-screening sensitivity while reducing clinically relevant overgrading. High-grade predictions in artefact-prone or inflamed regions should prompt targeted morphological examination rather than automatic grade escalation. In this way, the AI system may best retain value as a screening aid and quality-assurance tool.

## 7. Proposed Clinical Guardrails for Future Pathologist-Supervised Use

### 7.1. Core Safety Principles, Workflow Controls, and Deferral Criteria

The directional grading bias supports a cautious implementation strategy. The AI assigned a higher GG than ground truth in 47 of 109 malignant slides and a lower GG in only five. While weighted kappa values indicated substantial to high ordinal agreement, these metrics do not fully reflect the clinical significance of systematic overgrading. Initial evaluation is best conducted in a second-read or quality-assurance setting, with the pathologist recording an independent morphology-based interpretation before reviewing the AI output.

Neither a positive nor a negative AI result can abbreviate examination of the complete slide. The absence of false-negative predictions in this cohort does not establish equivalent performance in other populations, and a conspicuous or high-confidence mask does not confirm malignancy. Predicted Gleason pattern 4 or 5 regions require morphological verification, particularly when they are small, isolated, discontinuous, edge-associated, or overlap with artefact or inflammation. Such predictions should prompt targeted review rather than automatic upgrading.

Technical adequacy requires confirmation before interpretation of the AI output. Predictions arising in folded, crushed, poorly stained, incomplete, or out-of-focus regions can be unreliable and are better disregarded or deferred. Corrective action may include rescanning, recutting, restaining, preparing deeper levels, or completing conventional review without AI support. Detailed defect-specific actions are summarised in Table 9, and digital quality-control or image-harmonisation methods may support this process [24,25].

Histological mimics, including dense inflammation, lymphoid aggregates, atrophic or tangentially sectioned glands, stromal condensation, HGPIN, and ASAP, may also generate false-positive or high-grade predictions. Because HGPIN and ASAP were annotated as Gleason pattern 3 during development, an AI-positive focus does not establish invasive carcinoma. Equivocal regions require resolution using accepted morphological criteria, additional levels or cores, immunohistochemistry where indicated, or specialist review. The AI must not lower the diagnostic threshold or convert indeterminate morphology into carcinoma.

Substantial AI–pathologist discordance warrants activation of an escalation or deferral pathway. This includes high-grade predictions in tissue considered benign or low grade, predictions confined to artefact, low-confidence or internally conflicting outputs, and wide differences between AI-suggested and morphology-based GGs. Management may include targeted re-review, exclusion of unreliable masks, ancillary testing, genitourinary pathologist review, or withholding the AI-derived grade. No numerical discordance threshold was validated in this study; any proposed threshold requires prospective evaluation.

**Figure 4:**
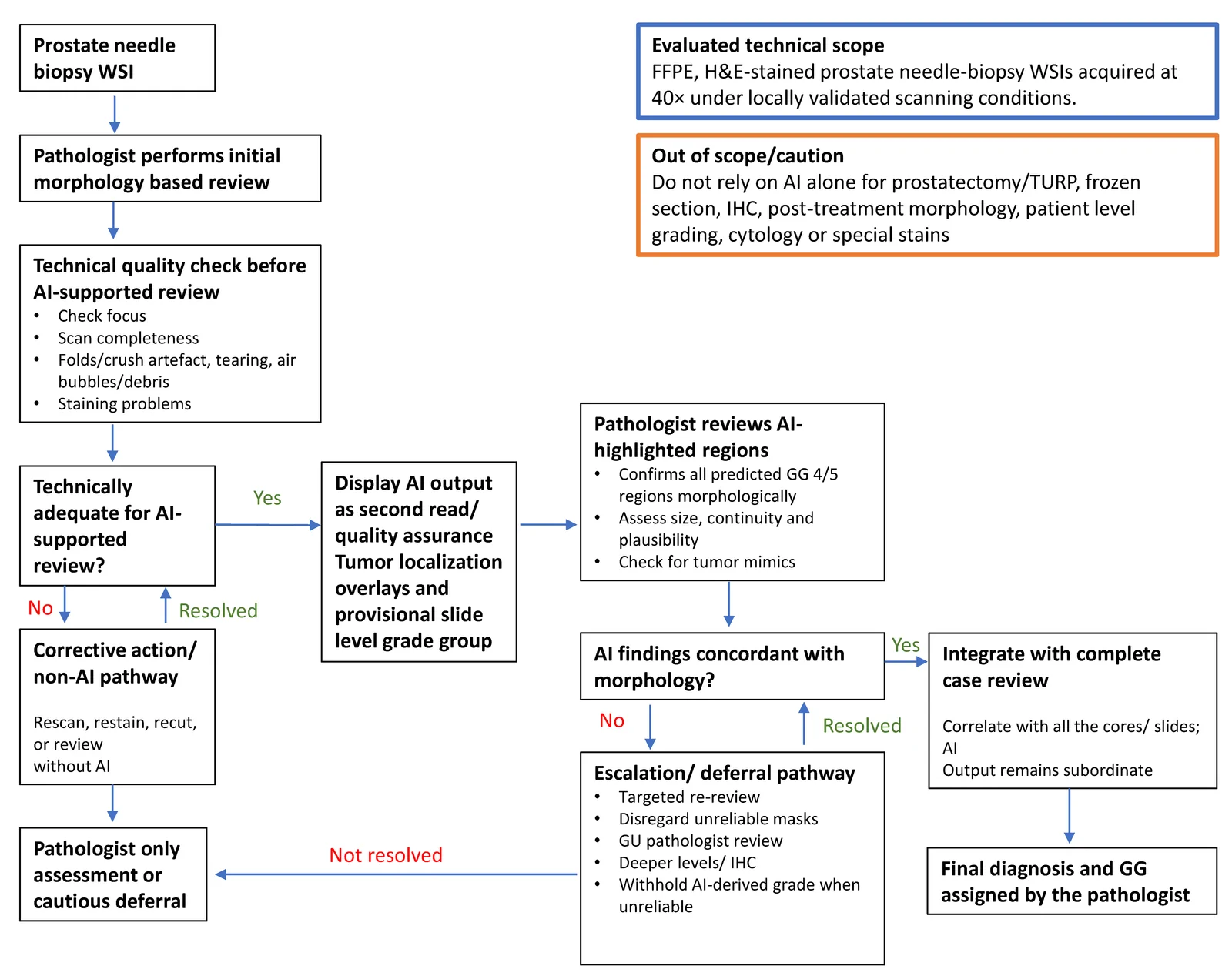
Proposed Clinical Guardrails for Future Pathologist-Supervised Use workflow for AI-supported review of prostate needle-biopsy whole-slide images highlighting the core safety principles, workflow controls, and deferral criteria. Technical adequacy and an independent morphology-based assessment precede AI review. Concordant findings are integrated with the complete case, whereas technically compromised, discordant, low-confidence, or out-of-scope outputs undergo corrective action, escalation, or deferral. Final diagnosis and ISUP GG assignment remain the responsibility of the reporting pathologist. The workflow requires prospective validation. Abbreviations: AI, artificial intelligence; FFPE, formalin-fixed, paraffin-embedded; GG, Grade Group; GU, genitourinary; H&E, haematoxylin and eosin; IHC, immunohistochemistry; ISUP, International Society of Urological Pathology; TURP, transurethral resection of the prostate; WSI, whole-slide image.

Use should remain restricted to formalin-fixed, paraffin-embedded, H&E-stained prostate needle-biopsy WSIs acquired under locally validated scanning conditions. Separate validation is required for other specimen types, post-treatment biopsies, special stains, immunohistochemistry, unfamiliar scanners, or materially altered laboratory workflows. The system does not separately identify cribriform architecture or intraductal carcinoma and was evaluated at slide rather than patient level. These and other required reporting features must be assessed independently and integrated across all cores, levels, and available clinical information (Figure 4).

Regional or slide-level deferral should be available when image quality is poor, morphology is out of scope, predictions are uncertain, or AI and pathologist assessments are substantially discordant. Explicit deferral is preferable to forcing an unreliable output and may reduce false reassurance or inappropriate reliance [27,28].

### 7.2. Pre-Deployment Local Validation and Pathologist Training

Before deployment, each laboratory needs to validate the system using slides prepared, stained, scanned, and reviewed under its intended conditions of use. Evaluation should extend beyond overall accuracy or kappa and include tumour-detection sensitivity, specificity, false-positive burden, the direction and magnitude of GG discrepancies, and clinically important upgrades or downgrades. The validation cohort should include low-volume carcinoma, borderline Gleason pattern 3 versus 4 morphology, artefact-rich slides, inflammation, benign mimics, and diagnostically equivocal foci.

Case mix must reflect the target population. The present test cohort contained only four GG 1 cases and was enriched for higher-grade disease. Laboratories serving active-surveillance populations or encountering more low-volume GG 1 and 2 tumours may experience different rates of false-positive detection and overgrading. Performance should be assessed separately across low-, intermediate-, and high-grade categories.

Acceptance criteria should be defined prospectively according to the intended workflow. Screening applications may prioritise sensitivity and negative predictive value, but an excessive false-positive burden could increase review time and alert fatigue. Grading support requires closer attention to systematic upgrading. Revalidation should follow material changes to tissue processing, staining, scanner hardware or software, image compression, preprocessing, model version, confidence thresholds, or slide-level aggregation. Site-specific calibration may be required when local performance differs from that observed in the development setting [26].

Pathologist training should include the validated scope, segmentation colour codes, known technical and histological failure modes, and the model’s tendency toward overgrading. Training also needs to include both correct predictions and visually convincing errors. The interface must preserve the primacy of the original H&E image, allow overlays to be toggled, and identify the regions contributing to the proposed GG. Delaying display of the AI grade or separating tumour localisation from grading may reduce anchoring, but these approaches require prospective assessment.

### 7.3. Post-Deployment Governance

Post-deployment governance should use continuous, auditable monitoring rather than one-time validation. Records should include the model version, scanner and staining conditions, quality-control status, AI output, final pathologist diagnosis, AI-pathologist discordance, whether the pathologist changed the diagnosis after AI review, and the reason for accepting or rejecting the AI suggestion. Periodic observations should assess model accuracy and identify performance drift, recurrent failure modes, changes in case mix, unexpected workload effects, the frequency of unnecessary secondary reviews, additional levels or immunohistochemistry prompted by false-positive outputs, reliance on high-grade suggestions, and alert fatigue. Particular attention is warranted for benign-to-malignant changes, upgrades from low- to high-risk GGs, and artefact- or inflammation-associated discordance. Predefined governance triggers could include rising false-negative detections, increased major overgrading, recurrent quality-control failures, or deterioration after scanner, staining, or model updates.

Discordant cases should feed into an iteration cycle. Regions falsely labelled as tumour or Gleason pattern 4 or 5 may be reused as hard-negative or borderline examples, provided that retraining is governed by rigorous annotation and independent validation. Updates need to avoid overfitting to a narrow local dataset or reducing sensitivity in an attempt to eliminate every false-positive prediction. The aim is to improve clinically meaningful discrimination while preserving the intended screening function.

Controlled prospective reader studies should compare unaided pathologist reporting, AI tumour-detection support without a GG suggestion, and full AI support with segmentation and grading output. Endpoints should include tumour-detection accuracy; exact and weighted GG concordance; the direction and magnitude of grading errors; interobserver agreement; reporting time; reader confidence; the frequency of diagnostic changes after AI review; the proportion of AI-induced changes that are correct or incorrect; deferral and secondary-review rates; and performance in artefact-rich or inflammatory cases. These measures would help determine whether the safeguards improve safety without removing the potential benefit of AI-assisted screening.

Studies also need to assess feasibility in resource-constrained environments, where universal subspecialist reporting, immediate immunohistochemistry, or repeated scanning may be impractical. Guardrails must use locally available escalation pathways while preserving a minimum safety principle: uncertain, artefact-affected, or morphologically discordant outputs must not be forced into definitive AI-generated diagnoses. Where specialist or ancillary resources are limited, explicit deferral and continued reliance on conventional morphological assessment may be safer than broadening the authority of the AI output.

Together, these guardrails form a layered safety framework extending beyond pathologist oversight to input-slide quality, the evidential status of AI outputs, sequencing of human and AI interpretation, management of discordant or uncertain cases, limits of the validated clinical scope, and ongoing institutional governance. They should preserve the system’s potential value for tumour screening and targeted review while reducing the risk that artefact-related false-positive or higher-grade predictions influence the final diagnosis without adequate morphological confirmation. Until the safeguards and associated AI-assisted workflow have been prospectively evaluated, the system should remain a pathologist-supervised screening and quality-assurance aid rather than an autonomous diagnostic or grading platform. The principal safeguards and recommended actions are summarised in Table 9.

## 8. Limitations

### 8.1. Study Design and Clinical Scope Limitations

The findings should be interpreted as evidence of encouraging slide-level performance in a controlled retrospective setting rather than routine clinical utility. Although the model performed strongly in benign-versus-malignant classification and showed some agreement with ground-truth ISUP GGs, its effectiveness in clinical practice has not been established. Limitations related to the absence of an AI-assisted reader arm, automation bias, and generalisability across laboratories and scanner platforms are addressed separately in Section 8.2 and Section 8.3.

Estimating time savings was not an objective of this study. The AI generated outputs within approximately 45 s to 1 min per WSI under controlled computational conditions, but technical inference time should not be equated with reduced pathologist reporting time. Real-world efficiency also depends on slide upload and image-transfer times, pre-processing, viewer integration, case loading, interpretation of AI overlays, user interaction, reporting behaviour, and familiarity with the system. The study demonstrates computational feasibility but provides no quantitative evidence of improved turnaround time or reporting efficiency.

The study was not designed as a prospective clinical validation. The AI was evaluated retrospectively using a predefined external test cohort and compared with an independently established ground truth. Although this design permits controlled assessment of diagnostic performance, it does not reproduce the complexity of real-time practice, in which cases vary in fixation, tissue processing, staining and image quality, case mix, reporting requirements, and available clinical information. Prospective studies are required to determine performance in routine diagnostic workflows.

The external test cohort also contained relatively few ISUP GG 1 cases. This distribution may reflect the regional clinical context, in which widespread prostate cancer screening is not routinely practised and patients may present with higher-grade or more clinically apparent disease. The small number of GG 1 cases limits conclusions regarding performance in low-grade carcinoma. Subsequent studies should include larger numbers of GG 1 cases, particularly small-volume and diagnostically subtle tumours, to assess sensitivity, grading accuracy, and the risk of overgrading in this clinically important category.

Evaluation was conducted at the level of individual WSIs rather than complete patient-level biopsy sets. Routine prostate biopsy diagnosis requires integration across multiple cores and deeper levels, together with tumour extent, laterality, percentage core involvement, perineural invasion, cribriform architecture, intraductal carcinoma, relevant clinical information, and previous biopsy history where available. Slide-level performance does not capture the full complexity of patient-level decision-making. Future studies should try to incorporate all biopsy cores from each patient and evaluate the system’s effect on final case-level reporting.

Post-treatment prostate biopsies were not included, including specimens obtained after hormonal therapy, radiation therapy, chemotherapy, or other treatment modalities. Treated tumours and treatment-related epithelial, stromal, and inflammatory changes may show altered morphology and can be challenging even for experienced pathologists. Performance in treated or post-therapy biopsies is therefore unknown, and the model must not be assumed to generalise to such material without dedicated validation.

The current algorithm does not separately detect or classify cribriform architecture, an adverse histological feature and important reporting element in prostate carcinoma. It also provides no separate output for intraductal carcinoma, although intraductal carcinoma was annotated as Gleason pattern 4 during algorithm development. More advanced versions should consider dedicated recognition of cribriform architecture and intraductal carcinoma because these features may independently influence prognosis, reporting, and clinical management.

The annotation strategy may also affect model behaviour. High-grade prostatic intraepithelial neoplasia and atypical small acinar proliferation were annotated as Gleason pattern 3 to prioritise sensitivity and ensure that potentially suspicious foci were highlighted for review. This approach is consistent with an assistive screening tool intended to reduce missed suspicious or malignant foci, but it may increase false-positive tumour predictions, particularly in cases containing limited atypical glands or borderline morphology.

Immunohistochemistry was not used during test-case evaluation. Expert GU pathologists established ground truth from haematoxylin and eosin morphology alone and achieved consensus without ancillary testing. In routine practice, however, immunohistochemistry may be required in selected equivocal cases, including those involving small atypical glands, borderline foci, assessment of basal-cell preservation, or difficult benign mimics. The performance of the AI system in cases requiring ancillary confirmation was not assessed.

Finally, the external test cohort was modest in size compared with some larger published validation studies. Broader validation is particularly important for rare or under-represented categories, including low-volume GG 1 carcinoma, post-treatment biopsies, cribriform architecture, intraductal carcinoma, and diagnostically equivocal lesions. Until such evidence is available, final diagnosis, ISUP GG assignment, recognition of clinically relevant histological features, and adjudication of false-positive outputs must remain the responsibility of the reporting pathologist. Prospective, workflow-integrated, multi-institutional studies are required to define the system’s appropriate role in routine clinical practice.

### 8.2. Lack of an AI-Assisted Reader Arm, Real-World Clinical Impact, and Automation Bias

The study evaluated the standalone performance of the AI system rather than its use by pathologists in an AI-assisted diagnostic workflow. While the AI output was compared with the unaided consensus interpretation of 11 pathologists against a ground truth established by GU pathologists, no dedicated AI-assisted reader arm was included. The findings could not show that AI assistance had a positive impact on the workflow by improving pathologist accuracy, reducing reporting time, increasing diagnostic confidence, or enhancing interobserver agreement in routine practice. They demonstrate that the algorithm can detect prostate carcinoma with high sensitivity and assign an ISUP GG under experimental conditions, but not how pathologists would use, accept, modify, or reject the segmentation overlays during clinical reporting.

Clinical value depends on human–AI interaction as well as standalone performance. The system could function as a first-read screening tool to prioritise suspicious cases, a second-read safety check to reduce missed malignancies, a triage tool for high-volume workloads, or a quality-assurance aid for identifying discordant or potentially undercalled regions. It was not within the scope of this study to determine the most appropriate use in clinical practice.

Automation bias remains an important but incompletely characterised risk in AI-assisted pathology. Segmentation overlays improve interpretability by showing the regions contributing to a prediction but can also influence judgement. Pathologists could place undue weight on incorrect visually convincing highlights, particularly when accompanied by a high-grade prediction. In this study, some discordant cases were overgraded when technical artefacts, inflammation, tissue folds, or limited tumour foci were incorrectly called higher ISUP GGs. Uncritical acceptance of these overlays could anchor readers toward a higher-grade interpretation. Conversely, failure to highlight a subtle malignant focus could provide false reassurance, although no such false-negative event occurred in the present validation cohort.

The findings do not establish clinical benefit in an integrated diagnostic workflow. Validation should use a prospective or controlled crossover reader-study design comparing unaided pathologist interpretation, standalone AI performance, and AI-assisted pathologist interpretation on the same case set. The design should include appropriate washout periods, randomised case order, pathologists with varying levels of experience, and predefined endpoints: tumour-detection accuracy, ISUP GG concordance, interobserver agreement, reporting time, diagnostic confidence, the frequency and direction of diagnostic changes after AI review, and the proportion of AI-induced changes that are correct or incorrect relative to expert ground truth.

AI-assisted workflow studies must also assess automation bias by documenting whether pathologists appropriately accept correct suggestions and reject incorrect ones. Discordant cases involving inflammation, artefacts, small-volume tumour, or borderline Gleason pattern 3 versus pattern 4 morphology require detailed review to determine whether AI support improves interpretation or introduces new errors.

### 8.3. Generalisability Constraints Related to Single-Biobank Sourcing and Single-Scanner Acquisition

The generalisability of the AI module is limited by the composition of the test cohort and by the pre-analytical and image-acquisition conditions under which it was evaluated. Each whole-slide image (WSI) was treated as an independent slide-level observation, with no more than two slides included from any biopsy case to reduce within-case duplication. The external test cohort comprised 150 diagnostically interpretable WSIs: 41 benign (27.3%) and 109 malignant (72.7%). GGs 4 and 5 accounted for 52.3% of malignant cases, whereas GG 1 was markedly under-represented.

This distribution introduces spectrum and prevalence effects. Higher-grade tumours are generally more extensive and morphologically conspicuous than small-volume GG 1 carcinoma, and their enrichment may produce favourable aggregate tumour-detection estimates while providing limited information about subtle low-grade disease. With only four GG 1 cases, sensitivity, exact grading agreement, and overgrading rates cannot be estimated reliably for this category. The high malignant prevalence may also increase the positive predictive value observed in the study; in laboratories with a lower cancer prevalence, the same sensitivity and specificity could generate a greater proportion of false-positive alerts and increase the number of benign slides requiring assessment.

Athough data was obtained through a single biobank, the submitted specimens originated from multiple contributing centres. Limiting the number of slides per case and retaining staining variability increased morphological diversity but did not create a fully independent multi-institutional validation cohort. Centralised case selection, common eligibility criteria, and acquisition on a single scanner may preserve correlated characteristics across the dataset. Consequently, the 150 WSIs represent fewer independent laboratory and imaging domains than their numerical size alone suggests, and performance may decline when the module encounters pre-analytical patterns not represented in the test cohort.

All slides were manually stained. Variation in haematoxylin intensity, eosin balance, contrast, background staining, and batch quality was accepted when the morphology remained diagnostically interpretable. Such variation can alter the colour and texture distributions used by convolutional models to identify glands, nuclei, stroma, and tumour architecture. Understaining may reduce nuclear and glandular contrast, potentially lowering tumour-detection confidence, whereas overstaining, precipitate, tissue folds, or colour imbalance may create high-frequency features that resemble poorly formed glands or high-grade tumour. These effects may increase false-positive segmentation, shift predicted Gleason patterns towards GGs 4 or 5 and produce unstable confidence scores. The artefact-associated false-positive and overgrading errors observed in this study are consistent with this type of input-domain sensitivity.

Conversely, testing on manually stained slides does not establish performance on automated staining platforms. Automated systems may produce more consistent slides but can have colour profiles and optical-density distributions that differ from those represented during model development. A model may therefore show calibration drift even when the slides remain readily interpretable to a pathologist. This means that the model may continue to rank malignant regions above benign regions, but scanner or stain-related shifts in confidence scores may render previously established classification thresholds unreliable, leading to false-positive or false-negative predictions.

All WSIs were acquired at 40× magnification using a single Grundium Ocus 40 scanner. This standardised resolution, colour processing, focus behaviour, compression, and image-tile generation, but prevented measurement of scanner-related domain shift. Differences in scanner optics, focal-plane selection, modulation transfer, colour calibration, compression, tissue-detection algorithms, and effective pixel resolution can change the histological appearance of the tissue by altering nuclear detail, gland contours, luminal structures, and stromal texture. These changes can affect tile eligibility, segmentation boundaries, Gleason-pattern probabilities, and the subsequent aggregation of tile-level outputs into a slide-level prediction. Even small shifts in confidence may move borderline cases across tumour-detection or GG thresholds, altering sensitivity, specificity, and grading concordance.

Differences in local case mix may further modify clinical performance. Laboratories that encounter more benign mimics, inflammation, small atypical acinar proliferations, low-volume carcinoma, or post-treatment specimens may experience a higher false-positive burden than that observed in this cohort. Prevalence differences will also alter positive and negative predictive values even when sensitivity and specificity remain unchanged. Aggregate accuracy from the present cohort should not be transferred directly to laboratories serving populations with substantially different disease prevalence or GG distributions.

Before deployment, the module needs to undergo local validation using slides prepared, stained, and scanned under the intended conditions of use. Evaluation should include tumour-detection sensitivity and specificity, false-positive burden, exact and weighted GG agreement, the direction of grading discrepancies, and performance in low-grade and artefact-enriched subsets. Confidence calibration should also be assessed after transfer to a new scanner or staining workflow, because the model’s confidence scores may no longer correspond to its actual probability of being correct, even when overall classification accuracy remains acceptable. Where performance differs materially, corrective action such as stain normalisation, scanner-specific calibration, revised confidence thresholds, or model retraining may be required.

The current findings therefore establish performance within a defined staining, scanner, and case-mix domain rather than universal portability. Multi-institutional validation across different scanners, staining platforms, tissue-processing protocols, and patient populations is required to determine whether the observed sensitivity, specificity, and GG agreement remain stable outside the study environment.

## 9. Conclusions

In this study, we evaluated an AI-based system for detecting prostate cancer and assigning ISUP GGs on prostate needle biopsy whole-slide images. The system demonstrated high sensitivity for benign-versus-malignant classification, with no false-negative tumour detections in the external test cohort and overall binary classification performance comparable to the multi-reader pathologist consensus. For ISUP GG assignment, the model demonstrated substantial to high ordinal agreement with GU pathologist-derived ground truth when weighted kappa metrics were applied, with many grading discrepancies limited to adjacent or near-adjacent categories.

These findings support further evaluation of the AI system as a pathologist-supervised screening aid for prostate biopsy assessment, with its main potential value in highlighting suspicious regions in high-volume diagnostic workflows. However, false-positive predictions, overgrading, artefact-related misclassifications, and the lack of dedicated outputs for cribriform architecture, intraductal carcinoma, and post-treatment morphology preclude autonomous diagnostic use. Final interpretation and ISUP GG assignment must remain the responsibility of the reporting pathologist.

The study also provides practical insight into the development and evaluation of pathology AI in a resource-constrained setting, where manual staining variability, limited access to ancillary testing, restricted scanner diversity, and reliance on cloud-based computation may reflect real-world implementation conditions. These features may be relevant to other groups developing AI tools in similar environments.

Further prospective, multi-institutional, multi-scanner, and workflow-integrated validation will be required to determine whether AI assistance improves diagnostic accuracy, reproducibility, reporting efficiency, pathologist confidence, and patient-level outcomes in routine practice. Until such evidence is available, the findings should be viewed as supportive of further evaluation of AI-assisted prostate biopsy screening, while preserving pathologist oversight as the cornerstone of final diagnosis.

## Figures and Tables

**Table 1:** Inclusion criteria used to choose the datasets used in the training, testing and validation of this AI module. Abbreviations: AI, artificial intelligence; FFPE, formalin-fixed, paraffin-embedded; H&E, haematoxylin and eosin; IRB, Institutional Review Board; WSI, whole-slide image.

Inclusion Criteria	
Specimen Type	1. Formalin-fixed, paraffin-embedded (FFPE) tissue sections 2. Prostate core needle biopsies 3. No more than two slides from a single case
Diagnostic adequacy	1. Slides deemed interpretable by a pathologist 2. Sufficient tissue present to render a diagnosis
Staining quality	1. Haematoxylin and eosin (H&E)–stained slides with acceptable contrast and colour balance 2. Variability in staining was permitted as long as the tissue was sufficient to render a diagnosis (to reflect real-world conditions)
Image quality	1. WSIs scanned at 40× magnification with adequate resolution. 2. Scanning artefacts, if present, must not interfere with diagnosis.
Temporal and institutional criteria	1. Slides collected between January 2021 and January 2022 2. Cases sourced from participating institutions under consistent protocols 3. Data were anonymised in accordance with the applicable IRB approval.
Artefacts	1. Slides with artefacts (e.g., folding, staining variation, minor scanning issues) may be included if they do not preclude diagnosis

**Table 2:** Exclusion criteria used to choose the datasets used in the training, testing and validation of this AI module. Abbreviations: AI, artificial intelligence; H&E, haematoxylin and eosin; WSI, whole-slide image.

Exclusion Criteria	
Non-diagnostic slides	1. Slides with insufficient tissue or poor preservation preventing diagnosis
Severe artefacts	1. Extensive tissue folding, tearing, crushing, or air bubbles obscuring key regions 2. Severe staining failure (e.g., over/understaining that obscures morphology)
Scanning/technical issues	1. Out-of-focus WSIs involving significant portions of the tissue affecting diagnosis 2. Incomplete scans or missing regions of interest 3. File corruption or unreadable formats
Non-representative or irrelevant samples	1. Non-prostate needle biopsy tissue 2. Cytology slides
Duplicate or redundant data	1. Duplicate slides from the same block
Special stains	1. Only H&E slides were used in this study

**Table 5:** Confusion matrix comparing ground truth (GT) and AI-predicted ISUP GG classifications. Abbreviations: AI, artificial intelligence; GG, Grade Group; GT, ground truth; ISUP, International Society of Urological Pathology.

	GT Benign	GT ISUP GG 1	GT ISUP GG 2	GT ISUP GG 3	GT ISUP GG 4	GT ISUP GG 5
AI Benign	38	0	0	0	0	0
AI ISUP GG 1	0	0	0	0	0	0
AI ISUP GG 2	1	2	5	3	0	0
AI ISUP GG 3	0	2	16	13	0	1
AI ISUP GG 4	0	0	1	0	3	1
AI ISUP GG 5	2	0	2	8	16	36

**Table 6:** Concordance in ISUP GG assignment was assessed using Cohen’s kappa, linearly weighted Cohen’s kappa, and quadratically weighted Cohen’s kappa. Abbreviations: AI, artificial intelligence; CI, confidence interval; GT, ground truth; ISUP, International Society of Urological Pathology. Values are Cohen’s κ with 95% CIs.

Metric	GT vs. AI
Cohen Kappa	0.53 (0.44–0.615)
Linear weighted Cohen Kappa	0.76 (0.68–0.82)
Quadratic weighted Cohen Kappa	0.87 (0.79–0.92)

**Table 9:** (**A**) Technical and image-quality artefacts affecting AI interpretation. The table summarises common slide-preparation, staining, scanning, and image-processing artefacts that may produce false tumour detection, erroneous Gleason pattern classification, or incomplete slide-level assessment. Recommended safeguards emphasise direct review of the underlying morphology, exclusion of unreliable regions, and corrective measures such as rescanning, restaining, recutting, or preparing deeper levels when appropriate. Abbreviations: AI, artificial intelligence; GG, Grade Group; WSI, whole-slide image. (**B**) Histological, interpretive, and governance-related safeguards. The table summarises histological mimics, discordant or uncertain AI outputs, use outside the validated scope, and post-deployment performance concerns that may result in false-positive classification, overgrading, false reassurance, or unreliable slide-level predictions. Recommended safeguards include morphological confirmation, review of additional levels or cores, ancillary testing or specialist assessment when indicated, structured deferral, and local audit, recalibration, revalidation, restriction, or suspension of the affected AI function. Abbreviations: AI, artificial intelligence; ASAP, atypical small acinar proliferation; HGPIN, high-grade prostatic intraepithelial neoplasia; ISUP, International Society of Urological Pathology; GG, Grade Group; GU, genitourinary.

**(A)**		
**Finding in the Slide or Image**	Potential AI Error	Recommended Safeguard
Tissue fold or crush artefact	Distorted benign tissue may be misclassified as tumour or Gleason pattern 4 or 5.	Review the underlying morphology without the overlay. Predictions confined to folded or crushed tissue must not determine the diagnosis or GG. Consider recutting or deeper levels when the affected area is diagnostically important.
Tissue tearing, fragmentation, or section-edge artefact	Irregular contours may produce false tumour segmentation or discontinuous high-grade masks.	Confirm that the prediction corresponds to intact, interpretable tumour architecture. Disregard unsupported edge-associated or fragmented masks and consider recutting when necessary.
Marked over- or understaining	Loss or exaggeration of morphological detail may cause false tumour detection or incorrect Gleason pattern classification.	Determine whether the morphology remains interpretable. Do not rely on the affected mask; consider restaining or preparing a new section when diagnostically relevant tissue is compromised.
Staining deposits, precipitate, or marked colour variation	Non-tissue material or altered colour characteristics may be interpreted as tumour or a higher Gleason pattern.	Exclude the affected region from AI-supported grading. Consider restaining, stain normalisation, or laboratory-specific calibration. Computational harmonisation must not compensate for uninterpretable morphology.
Air bubbles, coverslip defects, scanner debris, or pen marks	Spurious segmentation or false high-grade prediction.	Confirm whether the prediction overlaps the artefact. Clean, recoverslip, or rescan where feasible; otherwise, disregard the affected output.
Out-of-focus tissue	Unreliable gland detection, segmentation, or Gleason pattern classification.	Rescan the affected region or complete slide before interpreting the AI output. Negative or low-grade predictions from out-of-focus tissue should not be considered reliable.
Incomplete scan or missing tissue region	False-negative output or incomplete slide-level aggregation.	Confirm that all biopsy tissue has been captured and rescan before using the AI output. Do not accept a negative result when diagnostically relevant tissue is absent.
Image-stitching, compression, registration, or tile-boundary artefact	Discontinuous, duplicated, displaced, or distorted segmentation masks.	Compare the overlay directly with the original WSI. Disregard misaligned masks and repeat image acquisition or processing when the defect is extensive.
**(** **B** **)**		
**Finding in the Slide or AI Output**	Potential AI Error or Clinical Risk	Recommended Safeguard
Dense inflammation or lymphoid aggregates	False tumour detection or false Gleason pattern 4 or 5 prediction.	Confirm malignant glandular or cellular morphology. Assess cellular composition, glandular relationships, surrounding stroma, and continuity with unequivocal carcinoma where present.
Atrophic or tangentially sectioned glands, stromal condensation, HGPIN, or ASAP	False Gleason pattern 3 prediction or inappropriate diagnosis of invasive carcinoma.	Apply established morphological criteria and do not diagnose malignancy from segmentation alone. Use deeper levels, additional cores, immunohistochemistry, or specialist review when indicated.
Small, isolated, discontinuous, or edge-associated Gleason pattern 4 or 5 mask	Disproportionate increase in the slide-level ISUP GG.	Treat the mask as a review prompt rather than evidence for automatic upgrading. Consider its area, continuity, confidence, morphological plausibility, and overlap with artefact. Minimum-area or confidence thresholds should be used only after local validation.
High-confidence prediction within an artefact-prone region	False reassurance because the displayed model confidence is high.	Interpret confidence separately from diagnostic correctness. Confirm the original morphology and disregard unsupported predictions regardless of confidence score.
Negative AI output on a technically compromised slide	False reassurance or missed malignancy.	Confirm technical adequacy and complete tissue capture before considering the negative output supportive. The absence of an AI highlight must not abbreviate review of the complete slide.
AI-predicted GG substantially higher than the morphology-based assessment	Clinically important overgrading that may affect risk stratification or management.	Do not accept the AI grade as final. Re-examine the highlighted regions, exclude artefact-associated masks, review other cores or levels, and obtain GU pathologist review where appropriate.
Conflicting regional predictions or low-confidence output	Unstable or unreliable slide-level classification.	Use a structured deferral pathway rather than forcing a definitive AI category. Review the case morphologically and obtain additional investigations or specialist assessment where indicated.
Slide or specimen outside the validated technical or clinical scope	Unpredictable performance caused by distribution shift.	Do not rely on the output for unvalidated specimens, post-treatment material, special stains, immunohistochemistry, unfamiliar scanners, or substantially altered laboratory workflows. Complete separate validation before use.
Recurrent artefact-associated discordance after implementation	Performance drift or a systematic laboratory-, scanner-, or model-related failure mode.	Document and audit discordant cases, investigate technical causes, and reassess local performance. Consider recalibration, retraining, revalidation, workflow restriction, or temporary suspension of the affected AI function.

## Data Availability

All data generated or analysed during this study are included in this published article.
